# Understanding the high l-valine production in *Corynebacterium glutamicum* VWB-1 using transcriptomics and proteomics

**DOI:** 10.1038/s41598-018-21926-5

**Published:** 2018-02-26

**Authors:** Hailing Zhang, Yanyan Li, Chenhui Wang, Xiaoyuan Wang

**Affiliations:** 1 State Key Laboratory of Food Science and Technology, Jiangnan University, Wuxi, 214122 China; 2 School of Biotechnology, Jiangnan University, Wuxi, 214122 China; 3 Synergetic Innovation Center of Food Safety and Nutrition, Jiangnan University, Wuxi, 214122 China

## Abstract

Toward the elucidation of the advanced mechanism of l-valine production by *Corynebacterium glutamicum*, a highly developed industrial strain VWB-1 was analyzed, employing the combination of transcriptomics and proteomics methods. The transcriptional level of 1155 genes and expression abundance of 96 proteins were changed significantly by the transcriptome and proteome comparison of VWB-1 and ATCC 13869. It was indicated that the key genes involved in the biosynthesis of l-valine, *ilvBN*, *ilvC*, *ilvD*, *ilvE* were up-regulated in VWB-1, which together made prominent contributions in improving the carbon flow towards l-valine. The l-leucine and l-isoleucine synthesis ability were weakened according to the down-regulation of *leuB* and *ilvA*. The up-regulation of the branched chain amino acid transporter genes *brnFE* promoted the l-valine secretion capability of VWB-1. The NADPH and ATP generation ability of VWB-1 were strengthened through the up-regulation of the genes involved in phosphate pentose pathway and TCA pathway. Pyruvate accumulation was achieved through the weakening of the l-lactate, acetate and l-alanine pathways. The up-regulation of the genes coding for elongation factors and ribosomal proteins were beneficial for l-valine synthesis in *C. glutamicum*. All information acquired were useful for the genome breeding of better industrial l-valine producing strains.

## Introduction

*Corynebacterium glutamicum* (*C. glutamicum*) was isolated in 1957 by Kinoshita and coworkers in a screening program for l-glutamate producing bacteria from a soil sample collected at Ueno Zoo in Tokyo (Japan), it turned out to be a natural producer of L-glutamate under biotin limitation^1^. The nonpathogenic, GC-rich, Gram-positive bacterium has been used for over 50 years in the industrial production of l-amino acids because of its remarkable ability^2^. Besides the production of L-glutamate and l-lysine based on a large scale genetically modified high-performance strains^3,4^, respectively, *C. glutamicum* is also predominantly used in microorganism for the microbial synthesis of l-valine and other branched-chain amino acids (BCAA) (l-leucine and l-isoleucine)^5–7^. L-valine is an essential amino acid for vertebrates which is used as a feed additive and is a component of infusion solutions and cosmetics, it is also an important precursor in the chemical synthesis of herbicides^8^. In order to improve the efficiency of the l-valine production and other products of *C. glutamicum*, it is important to understand the composition and regulation of the metabolic pathways leading to these industrially important products. Hence, the whole genome sequencing and annotation of the type strain *C. glutamicum* ATCC 13032 and the wild-type strain *C. glutamicum* R have deeply influenced the bacterial fermentation performance promotion during the last few years^9–11^. The genome sequence knowledge also paved the way for the foundation of emerging genome-wide analysis techniques in fields of transcriptomics and proteomics^12–15^. While previous studies focused on specific genes or enzymes to enhance the l-valine biosynthesis ability of *C. glutamicum* from the viewpoint of carbon metabolic flux enhancement^8,16,17^, branched-chain amino acid transportation^18^ and cofactors supply project^19^. All those methods have played a certain role in the process of improving the yield of l-valine, however, the regulation of l-valine metabolism in *C. glutamicum* is a much more complex process. Here we present a global analysis of a high l-valine production *C. glutamicum* strain by the combination of the two advanced techniques, transcriptional profiling and two-dimensional gel electrophoresis, which allows us to fully understand the deep mechanism of l-valine biosynthesis and other cell growth and metabolism regulation type correlated to it.

## Results

### Comparison of growth and amino acid production, proteome and transcriptome differences between *C. glutamicum* VWB-1 and ATCC 13869

**Figure 1:**
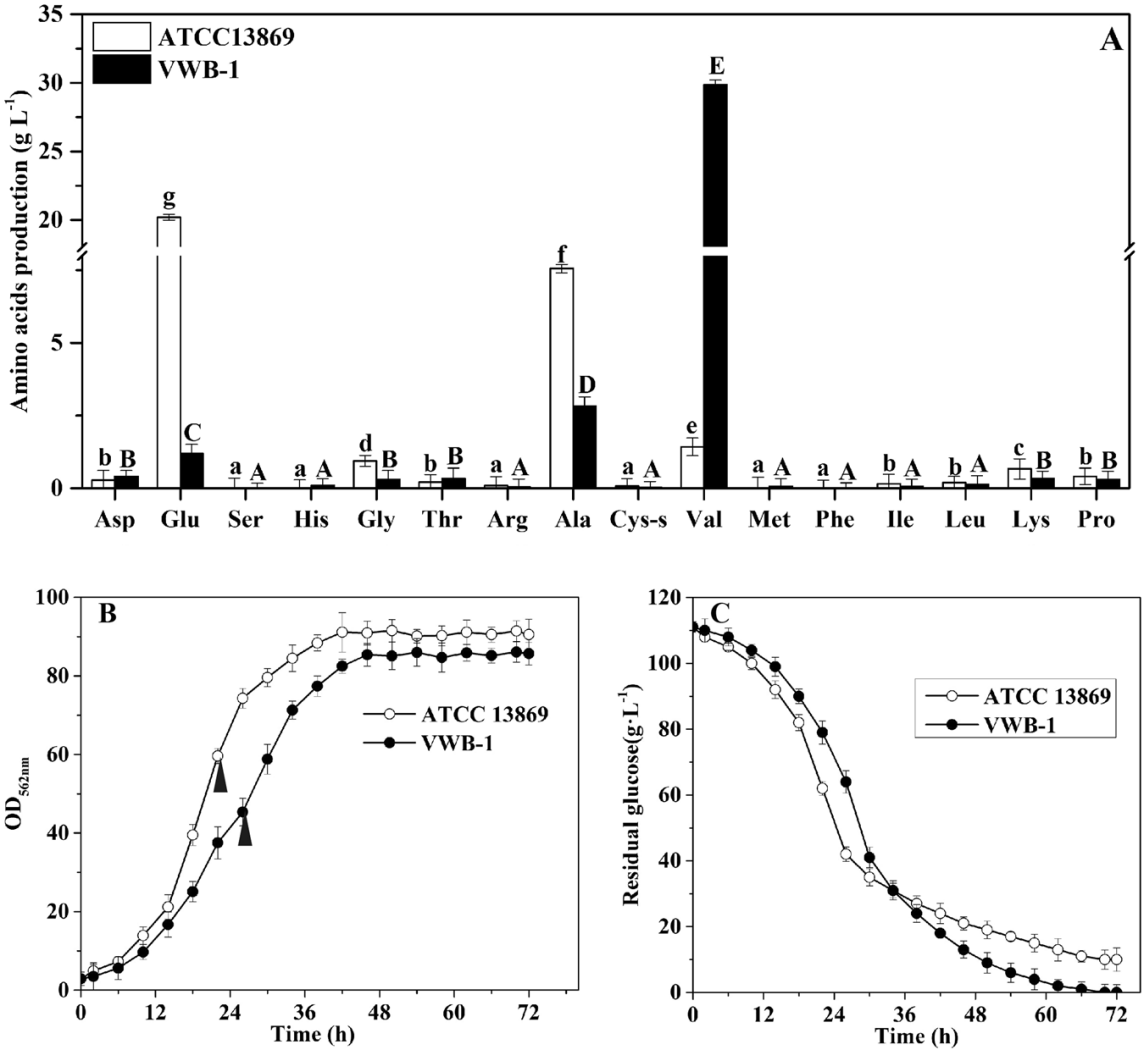
Comparison of amino acids levels (**A**), biomasses (**B**), and residual glucose (**C**) in batch fermentation between *C. glutamicum* VWB-1 and ATCC 13869. The cell harvest point for omics analysis are indicated by black arrows. Ala, L-alanine; Glu, L-glutamate; Arg, L-arginine; Pro, L-proline; Asp, L-aspartate; Lys, L-lysine; Thr, L-threonine; Met, L-methionine; Ile, L-isoleucine; Val, L-valine; Leu, L-leucine; Ser, L-serine; His, L-histidine; Gly, L-glycine; Tyr, L-tyrosine; Cys-s, L-cysteine; Phe, L-phenylalanine. Error bars indicate the standard deviations from three parallel samples. (a–g) and (**A**–**E**) represent the statistically significant differences (g > f > e > d > c > b > a; E > D > C > B > A).

**Figure 2:**
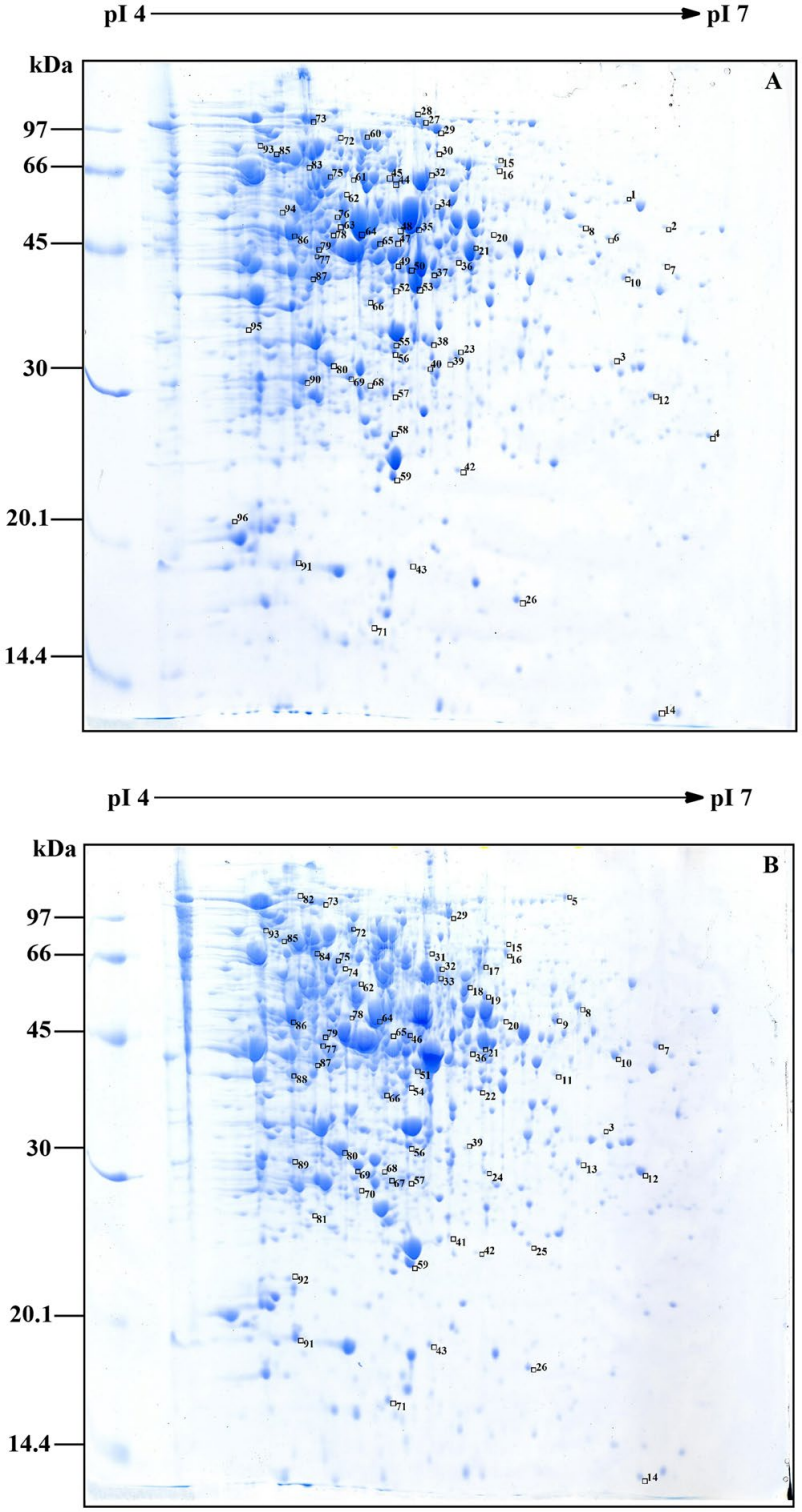
2-DE comparison of the total proteins from *C. glutamicum* VWB-1 (**A**) and ATCC 13869 (**B**). Boxes from the top right corner of the protein spots represent the expression up-regulation; boxes from the bottom right corner of the protein spots represent the expression down-regulation. Original gel scan image of (**A**) and (**B**) were provided in Figure S1.

**Figure 3:**
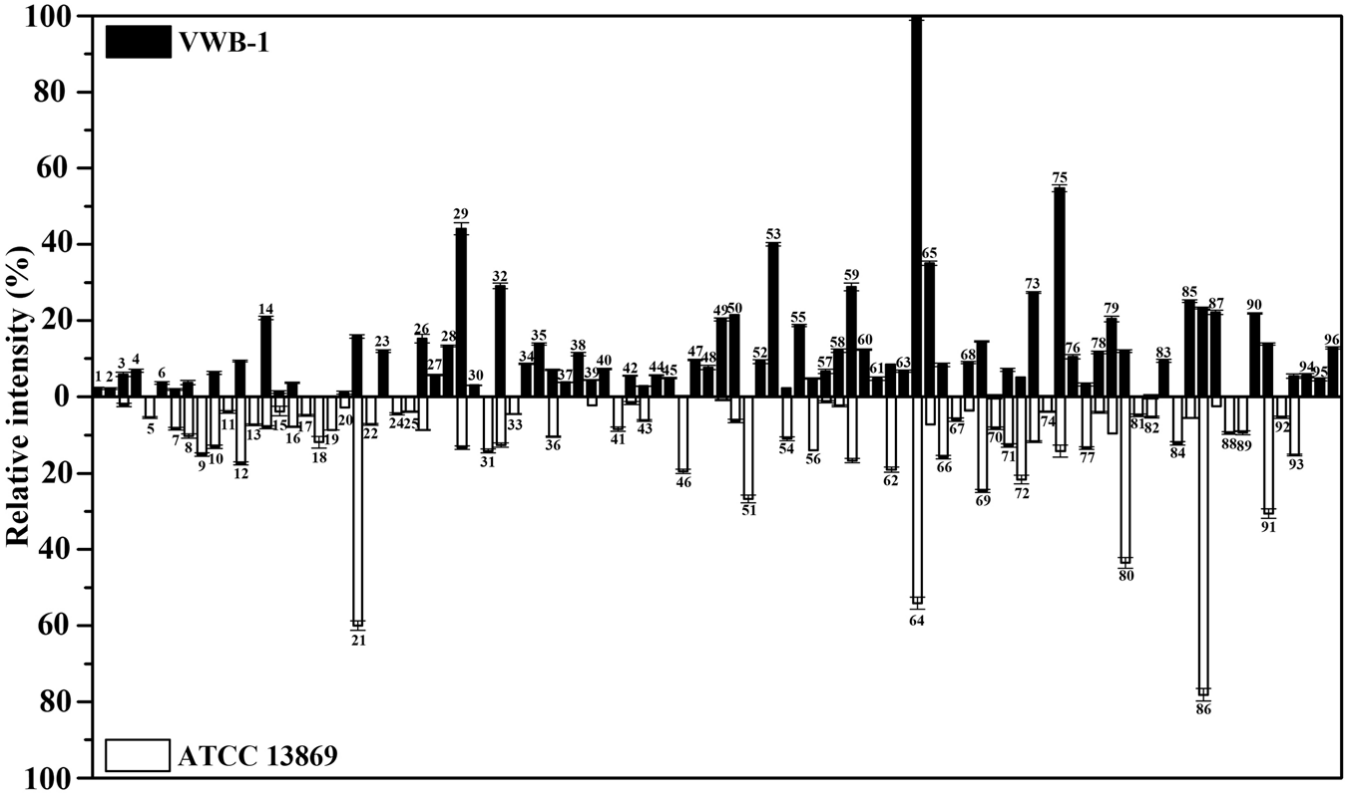
Relative expression levels of differentially expressed proteins identified by proteomic analysis. The number on the columns were corresponding to the protein spots number in Fig. 2(A) and (B). Error bars stand for the standard deviation.

**Figure 4:**
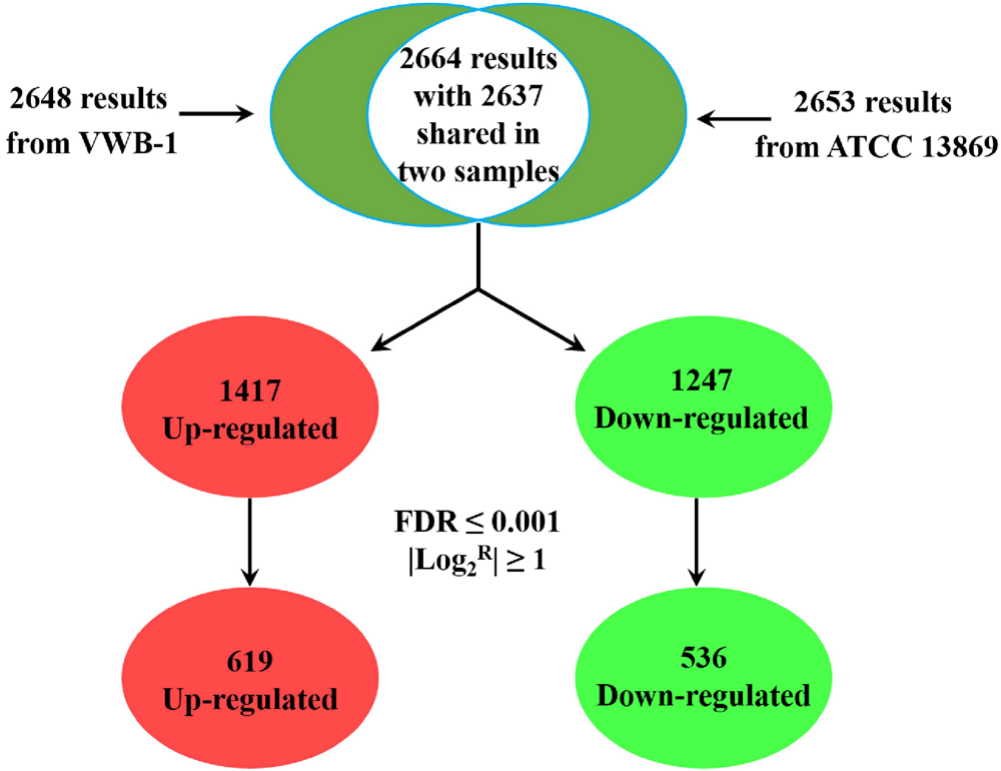
Mathematical statistics on the number of DEGs between *C. glutamicum* VWB-1 and ATCC 13869. FDR stands for the false discovery rate; log_2_^R^ represents the ratio of gene transcription level (RPKM) between *C. glutamicum* VWB-1 and ATCC 13869.

*C. glutamicum* VWB-1 is a l-valine high-yield strain screened by multiple rounds of random mutagenesis, which led to the differential regulation patterns between VWB-1 and ATCC 13869, both in transcription and translation level. During the 72 h fed-batch fermentation experiment (Fig. 1), the two strains showed different characteristics in cell growth, glucose consumption and amino acid production. After a short period of lag phase, both strains entered the logarithmic phase in which ATCC 13869 grew faster than VWB-1, suggesting the existence of unfriendly regulation for growth in VWB-1. At the same time, the consumption rate of glucose was also increased, accompanied by the acceleration of cell growth and various metabolic activities. The glucose utilization of VWB-1 was slower than ATCC 13869 at the beginning, and later it surpassed that of ATCC 13869 with the arrival of late logarithmic and stationary phase, this might be due to the larger production of amino acids, especially the production of l-valine, largely increased the physiological demand of glucose. The composition and content of amino acids in the fermentation broth was analyzed by HPLC (Fig. 1A). The l-valine production was 29.85 g·L^−1^ in VWB-1, much higher than its original 1.42 g·L^−1^ yield in ATCC 13869. The production reduction of glutamate may help explaining the l-valine production increase in VWB-1, since it is the primary amino donor in l-valine biosynthesis. The l-valine synthesis capability enhancement is bound to more substrate requirement, pyruvate especially, however, it is interesting that the production of alanine, a branched pathway of l-valine biosynthesis which also applies pyruvate as a substrate, was lower in ATCC 13869 than in VWB-1. The production of other amino acids, such as glycine, lysine and proline were also decreased in VWB-1, the contents of the rest amino acids were relatively low. The multiple regulation which not only promoted the carbon metabolism to valine but also lowered down the yields of other amino acids makes VWB-1 an ideal workhorse for l-valine production. Proteome analysis applying the two-dimensional gel electrophoresis (2DE) technique which separates proteins by molecular charge and molecular size allows us to compare the actual amount of proteins present under certain environment conditions. As compared to ATCC 13869, the expression of 96 proteins in VWB-1 were changed significantly according to the proteomics results (Figs 2 and 3), 50 of all these differentially expressed proteins (DEPs) certified by TOF-MS were up-regulated and the other 46 were down-regulated, as shown in Table 1, these DEPs were classified according to the ways they participating in the cellular metabolism. While transcriptomics reflects the overall abundance of mRNA in a specific growth period, here, mRNA serves as a transient intermediary molecule in the information network. With the employment of high-throughput sequencing technique, the transcriptome difference between VWB-1 and ATCC 13869 was revealed, the transcriptional regulation of 1155 DEGs (shown in Fig. 4) covered from the biosynthetic pathways, cell metabolism, transcriptional regulators and so forth. The multiple regulation which not only promoted the carbon metabolism to l-valine but also lowered down the yields of other amino acids makes VWB-1 an ideal workhorse for l-valine production.

**Table 1:** Protein expression variation between *C. glutamicum* VWB-1 and ATCC 13869.

Spot	Alt. GeneID	GeneID	Gene Name	Annotation	pI/Mw	VWB-1	ATCC 13869	mRNA
**Carbohydrate metabolism**								
5	*NCgl1084*	*cg1280*	*kgd*	2-oxoglutarate dehydrogenase	5.92/138757.0	0.00	800.97	NC
6	*NCgl0658*	*cg0790*	*lpdA*	Flavoprotein disulfide reductase	5.61/49540.59	536.62	0.00	NC
7	*NCgl2817*	*cg3227*	*lldA*	L-lactate dehydrogenase	5.72/45714.48	278.17	1249.10	NC
13	*NCgl0960*	*cg1139*	—	Allophanate hydrolase subunit 2	7.93/31668.91	0.00	1094.03	2.4
15	*NCgl0360*	*cg0446*	*sdhA*	Succinate dehydrogenase A	5.37/74679.26	183.01	555.53	NC
16	*NCgl0360*	*cg0446*	*sdhA*	Succinate dehydrogenase A	5.37/74679.26	546.35	1167.85	NC
18	*NCgl2521*	*cg2891*	*poxB*	Pyruvate dehydrogenase	5.34/61950.39	0.00	1770.94	−8.9
21	*NCgl0795*	*cg0949*	*gltA*	Titrate synthase	5.19/48929.41	2369.38	8992.08	4.4
23	*NCgl2476*	*cg2836*	*sucD*	Succinyl-CoA synthetase subunit alpha	5.31/30259.75	1792.09	0.00	53.2
29	*NCgl2167*	*cg2466*	*aceE*	Pyruvate dehydrogenase subunit E1	5.25/102826.2	6609.52	1984.81	NC
35	*NCgl0355*	*cg0441*	*lpd*	Dihydrolipoamide dehydrogenase	5.41/50651.69	2076.01	0.00	ND
39	*NCgl1202*	*cg1409*	*pfkA*	6-phosphofructokinase	5.47/37088.16	657.44	336.87	NC
49	*NCgl1526*	*cg1791*	*gap*	Glyceraldehyde-3-phosphate dehydrogenase	5.16/36045.54	3039.63	136.57	−8.3
50	*NCgl1526*	*cg1791*	*gap*	Glyceraldehyde-3-phosphate dehydrogenase	5.16/36045.54	3209.93	938.70	−8.3
51	*NCgl1526*	*cg1791*	*gap*	Glyceraldehyde-3-phosphate dehydrogenase	5.16/36045.54	0.00	4006.09	−8.3
53	*NCgl2709*	*cg3107*	*adhA*	Zn-dependent alcohol dehydrogenase	5.23/36811.85	5992.18	0.00	66.7
61	*NCgl0670*	*cg0802*	*accBC*	Biotin carboxylase and carboxyl carrier protein	5.02/63419.65	710.24	0.00	5.1
63	*NCgl2698*	*cg3096*	*ald*	Aldehyde dehydrogenase	4.96/55106.23	1004.77	0.00	24.0
64	*NCgl0967*	*cg1145*	*fumC*	Fumarate hydratase	5.06/49763.37	14985.21	8104.40	6.8
68	*NCgl1856*	*cg2115*	*sugR*	Transcriptional regulators of sugar metabolism	5.03/27558.29	1349.13	525.30	3.9
82	*NCgl0802*	*cg0957*	*fas-IB*	Fatty acid synthase	4.74/315154.1	0.00	780.36	1.6
44	*NCgl2774*	*cg3179*	*fadD2*	Acyl-CoA synthase	4.66/67915.14	843.39	0.00	4.3
17	*NCgl2216*	*cg2521*	*fadD15*	Long-chain fatty acid CoA ligase	5.45/67060.39	0.00	715.35	−3.2
86	*NCgl0935*	*cg1111*	*eno*	Phosphopyruvate hydratase	4.65/44949.37	3486.36	11714.95	NC
94	*NCgl1396*	*cg1643*	*gnd*	6-phosphogluconate dehydrogenase	4.69/52579.02	851.76	0.00	1.6
**Specific biosynthesis pathways**								
1	*NCgl0578*	*cg0699*	*guaB2*	Inositol-monophosphate dehydrogenase	5.99/53360.95	321.74	0.00	NC
2	*NCgl2586*	*cg2964*	*guaB1*	Inositol-monophosphate dehydrogenase	6.39/50818.67	319.71	0.00	NC
4	*NCgl1025*	*cg1218*	*ndnR*	ADP-ribose pyrophosphatase	6.30/24013.42	1021.06	0.00	41.8
10	*NCgl2515*	*cg2885*	*bioA*	8-amino-7-oxononanoate transaminase	6.16/45740.64	939.16	1951.99	NC
11	*NCgl0562*	*cg0680*	*alr*	Alanine racemase	5.58/39173.67	0.00	586.78	1.7
19	*NCgl0875*	*cg1040*	—	ATPase component of ABC transporters	4.83/67200.96	0.00	1295.30	2.6
25	*NCgl0368*	*cg0454*	—	TetR family transcriptional regulator	5.41/24817.09	0.00	571.02	NC
32	*NCgl1219*	*cg1432*	*ilvD*	Dihydroxy-acid dehydratase	5.18/64674.11	4354.80	1888.03	2.8
33	*NCgl1219*	*cg1432*	*ilvD*	Dihydroxy-acid dehydratase	5.18/64674.11	0.00	667.12	2.8
34	*NCgl2930*	*cg3362*	*trpCF*	Indole-3-glycerol phosphate synthase	5.15/50477.16	1290.96	0.00	3.4
38	*NCgl1023*	*cg1215*	*nadC*	Nicotinate-nucleotide pyrophosphorylase	5.22/29376.34	1673.42	0.00	18.5
71	*NCgl1345*	*cg1585*	*argR*	Arginine repressor	5.08/18468.02	1055.88	1897.46	2.5
75	*NCgl1222*	*cg1435*	*ilvB*	Acetolactate synthase 1 catalytic subunit	4.82/66845.85	8202.69	2124.91	28.6
81	*NCgl2516*	*cg2886*	*bioD*	Dithiobiotin synthetase	4.65/23855.28	0.00	718.74	3.1
87	*NCgl2123*	*cg2418*	*ilvE*	Branched-chain amino acid aminotransferase	4.82/41691.16	3317.13	373.36	5.9
46	*NCgl2020*	*cg2304*	*hisC*	Probable histidinol-phosphate aminotransferase	4.85/39918.29	0.00	2920.91	NC
67	*NCgl2013*	*cg2297*	*hisF*	Imidazole glycerol phosphate synthase subunit	5.02/27244.85	0.00	891.29	−2.0
76	*NCgl2148*	*cg2447*	*glnA2*	Glutamine synthetase 2	4.86/50381.66	1574.11	0.00	4.2
77	*NCgl0237*	*cg0294*	*aspB*	Aspartate aminotransferase	4.85/46518.63	478.18	2004.65	NC
79	*NCgl2272*	*cg2586*	*proA*	Gamma-glutamyl phosphate reductase	4.77/45666.77	3057.61	1424.90	6.0
**Macroelement and metal homeostasis**								
37	*NCgl0120*	*cg0156*	—	Crp family regulatory proteins	5.29/40102.42	566.47	0.00	2.0
48	*NCgl2715*	*cg3114*	*cysN*	Sulfate adenyltransferase subunit 1	5.08/46871.63	1150.85	0.00	−1.6
58	*NCgl2943*	*cg3375*	—	Predicted nucleoside-diphosphate-sugar epimerase	5.19/22803.57	1811.41	357.77	4.6
89	*NCgl1875*	*cg2136*	*gluA*	Glutamate uptake system ATP-binding protein	5.67/26537.86	0.00	1402.16	8.6
70	*NCgl2482*	*cg2842*	*phoU*	Phosphate uptake regulator	5.11/28130.03	0.00	1231.04	NC
90	*NCgl2518*	*cg2888*	*phoR*	Two component response regulator	4.69/26349.95	3272.23	0.00	12.6
**SOS and stress response**								
8	*NCgl1490*	*cg1746*	—	Putative membrane protein	7.79/50850.63	558.75	1523.78	ND
31	*NCgl0251*	*cg0310*	*katA*	Catalase	5.18/58708.85	0.00	2119.04	3.3
45	*NCgl0251*	*cg0310*	*katA*	Catalase	5.18/58708.85	733.63	0.00	3.3
47	*NCgl1502*	*cg1763*	*sufD*	Components iron-regulated ABC-type transporter	5.16/42280.10	1443.52	0.00	1.8
60	*NCgl2682*	*cg3079*	*clpB*	ATP-dependent protease	5.00/93231.58	1852.55	0.00	NC
65	*NCgl1503*	*cg1764*	*sufB*	Component iron-regulated ABC-type transporter	4.85/53492.94	5255.30	1074.54	NC
**None module**								
3	*NCgl1529*	*cg1794*	—	Uncharacterised P-loop ATPase protein	6.01/34710.37	877.68	329.25	2.4
9	*NCgl0335*	*cg0412*	—	Membrane protein	4.98/40789.73	0.00	2259.05	−4.1
12	*NCgl2678*	*cg3073*	*sseA1*	Thiosulfate sulfurtransferase	5.67/29868.54	1402.17	2611.50	−3.4
14	*NCgl2881*	*cg3308*	*rpsF*	30S ribosomal protein S6	6.29/10958.78	3095.29	1181.77	NC
20	*NCgl0187*	*cg0238*	—	L-gulonolactone oxidase	5.68/53062.18	154.17	434.86	−3.0
22	*NCgl2620*	*cg3007*	*pvdS2*	RNA polymerase sigma factor, putative	5.31/35304.59	0.00	1072.35	−2.0
24	*NCgl1697*	*cg1990*	—	NUDIIX hydrolase	6.19/29557.92	0.00	655.30	
26	*NCgl0468*	*cg0572*	*rplJ*	50S ribosomal protein L10	5.63/17956.53	2285.72	1291.39	10.2
27	*NCgl0098*	*cg0129*	*putA*	Proline dehydrogenase	5.22/126322.0	857.19	0.00	5.3
28	*NCgl0098*	*cg0129*	*putA*	Proline dehydrogenase	5.22/126322.0	2001.23	0.00	5.3
30	*NCgl1900*	*cg2166*	*gpsI*	Polynucleotide phosphorylase/polyadenylase	5.04/81271.48	445.27	0.00	2.3
36	*NCgl2285*	*cg2601*	—	Pirin-related protein-fragment, partial	5.78/14160.96	1056.04	1554.78	−1.7
40	*NCgl1334*	*cg1573*	*tsnR*	23S ribosomal RNA methyltransferase	5.33/29989.91	1085.36	0.00	NC
41	*NCgl1754*	*cg2050*	—	Hypothetical protein	6.15/22196.02	0.00	1264.34	ND
42	*NCgl1920*	*cg2186*	—	Hypothetical protein	6.15/26633.53	830.10	247.13	2.9
43	*NCgl0612*	*cg0739*	—	Putative integral membrane protein	9.89/18278.42	413.77	917.71	2.0
52	*NCgl0647*	*cg0779*	*trpS*	Tryptophanyl-tRNA synthetase	5.11/37826.54	1378.57	0.00	NC
54	*NCgl0079*	*cg0109*	—	Triacylglycerol lipase precursor	5.11/35814.40	341.27	1637.98	NC
55	*NCgl2156*	*cg2456*	—	Zn-ribbon protein	5.19/26131.12	2802.33	0.00	2.7
56	*NCgl0317*	*cg0391*	*rmlB2*	dTDP-glucose 4,6-dehydratase	5.12/33536.53	717.37	2083.02	NC
57	*NCgl2359*	*cg2686*	—	TetR family regulatory protein	5.08/27672.61	999.65	195.64	−5.0
59	*NCgl1557*	*cg1825*	*efp*	Translation elongation factor P	5.14/20640.23	4319.67	2493.46	1.7
62	*NCgl0003*	*cg0005*	*recF*	DNA repair and genetic recombination protein	6.10/43185.22	1268.78	2845.32	NC
66	*NCgl0982*	*cg1164*	*ispH*	Penicillin tolerance protein	5.02/35602.44	1249.36	2364.57	NC
69	*NCgl0390*	*cg0482*	*gpmA*	Phosphoglyceromutase	4.91/27245.55	2173.02	3689.50	NC
72	*NCgl1255*	*cg1479*	*glgP1*	Glycogen phosphorylase	4.91/90494.91	751.29	3238.52	NC
73	*NCgl2068*	*cg2359*	*ileS*	Isoleucine-trna ligase-like protein	4.77/117403.9	4098.11	1747.63	15.6
74	*NCgl2594*	*cg2974*	*lysS*	Lysyl-tRNA synthetase	4.79/58926.83	0.00	574.26	NC
78	*NCgl2082*	*cg2373*	*murF*	D-alanine:D-alanine-adding enzyme	5.21/53265.99	1736.74	595.09	NC
80	*NCgl1949*	*cg2221*	*tsf*	Translation elongation factor ts (EF-Ts)	4.90/29279.98	1793.80	6521.77	2.1
83	*NCgl2219*	*cg2527*	*dcp*	Peptidyl-dipeptidase A protein	4.70/73880.16	1408.44	0.00	NC
84	*NCgl2206*	*cg2511*	—	Membrane protein containing CBS domain	4.66/47768.97	0.00	1818.35	3.4
85	*NCgl2217*	*cg2523*	*malQ*	4-alpha-glucanotransferase	4.75/78524.38	3756.57	815.02	−1.9
88	*NCgl0884*	*cg1051*	—	Hypothetical protein	4.96/43075.70	0.00	1413.45	2.3
91	*NCgl0884*	*cg0870*	—	Haloacid dehalogenase/epoxide hydrolase family	4.29/14458.40	2069.05	4579.75	−3.1
92	*NCgl2659*	*cg3050*	—	Acyltransferase	4.61/20172.76	0.00	790.25	−1.5
93	*NCgl0726*	*cg0868*	*secA*	Preprotein translocase subunit seca	5.01/95404.70	809.37	2279.75	NC
95	*NCgl0336*	*cg0413*	*cmt1*	Trehalose corynomycolyl transferase	4.56/39506.27	683.01	0.00	3.0
96	*NCgl0888*	*cg1055*	*menG*	ribonuclease activity regulator protein RraA	4.53/17364.56	1918.89	0.00	10.9

Accession numbers according to the NCBI database. Molecular mass (MW) and isoelectric point (pI) correspond to the NCBI database. Proteins showed two or more-fold changes in their expression pattern in all biological and technical replicates were regarded as regulated. The ration refers to the relative volume of the corresponding protein spot in continuous versus batch mode of fermentation. As for transcriptional regulation type of the corresponding gene, the qualitative results are indicated with the marks “Up” (ratio above 2.0), “Down” (ratio blow −2.0), and “NC” (not changed, 2.0 > ration > −2.0) in the ration column.

### Proteome analysis in specific biosynthesis pathways and carbohydrate metabolism

Protein spots with different gray level correlated with l-valine biosynthesis were detected and identified. The three key enzymes involved in the anabolism of l-valine in *C. glutamicum*, the catalytic subunit of acetohydroxy acid synthase (AHAS), the dihydroxy-acid dehydratase (DHAD) and the branched-chain amino acid transaminase (BCAT), separately encoded by *ilvB*, *ilvD*, and *ilvE*, were all up-regulated in VWB-1, they all play essential roles in guiding the carbon metabolic flow to l-valine, thus contributing directly to the yield promotion of l-valine.

The expression intensity of many enzymes that catalyze the branch metabolic pathway of l-valine synthesis had been altered. For instance, the expression quantity of D-alanine racemase that converts l-alanine to D-alanine was down-regulated in VWB-1, as alanine biosynthesis pathway is the substrate competition branch of l-valine synthesis and D-alanine is the primary hetero amino acids of l-valine production, this down-regulation of the protein encoded by *alr* might lead to the carbon flow diversion reduction and by-product formation decrease, thus not only brought the l-valine production increase, but also cut the downstream purification cost. As for the enzymes involved in other amino acids biosynthesis, the protein expression variation can also be observed. The aspartate aminotransferase encoded by *aspB* was down-regulated in VWB-1, resulting the low conversion efficiency between aspartate and l-glutamate. Transcriptional regulator of the arginine metabolism, the arginine repressor ArgR, was down-regulated in VWB-1, it regulates the arginine biosynthesis when combined with arginine by binding the site that overlap the promoter of the arginine biosynthesis genes, including *argBCD*, *argFGH* and *argJ*, whose transcriptional level were all up-regulated in VWB-1, leading to the positive regulation of arginine biosynthesis. However, the arginine production of VWB-1 was actually lower than that of ATCC 13869, this might be due to the reason that l-glutamate is not only the precursor of arginine biosynthesis but also the amino donor in l-valine anabolism, the biosynthesis of l-valine has cost most of the l-glutamate generated in VWB-1. Therefore, although the genes involved in arginine biosynthesis showed some extent of up-regulation in their transcriptional level, the accumulation of arginine showed a decrease in VWB-1 due to the lacking of precursor in its biosynthesis process. The tryptophan biosynthesis ability of VWB-1 was also promoted because of its up-regulated monomeric bifunctional protein (encoded by *trpCF*), combined with the mRNA level up-regulation of all the genes involved in this pathway, *trpA-G* (later mentioned). In addition, the histidinol-phosphate aminotransferase (*hisC* encoded) and the imidazole glycerol phosphate synthase subunit (*hisF* encoded) were both down-regulated in the l-histidine biosynthesis pathway.

As the substrate of l-valine biosynthesis, the generation and consumption of pyruvate are of great importance in l-valine production. Pyruvate is a central intermediate of *C. glutamicum*, it represents the turntable distributing unit which control the metabolic interconversion between glucose, fatty acid and amino acids through acetyl-CoA, citric acid cycle and other means. Pyruvate is also used for regeneration of NAD^+^ by the formation of lactate under anaerobic conditions. Enzymes catalyzing the reactions employing pyruvate as a substrate or product are working as pyruvate switches. The expression of pyruvate dehydrogenase (encoded by *poxB*), which catalyzes the formation of acetate from pyruvate, was down-regulated in VWB-1. Another important enzyme, the pyruvate dehydrogenase complex (PDHC), is a major pyruvate converting enzyme in *C. glutamicum*, which represents an attractive target for metabolic engineering. The *aceE* encoded pyruvate dehydrogenase subunit E1 component was up-regulated in VWB-1, PDHC E1 catalyzes the reaction of pyruvate decarboxylation to form acetyl-CoA, an essential source to feed the TCA cycle and thereby to satisfy the cellular requirements for the precursor metabolites it forms. Other proteins correlated to the pyruvate metabolism were two L-lactate dehydrogenases (LDHs), separately encoded by *lldA* and *ldh*, their combined down-regulation resulted in the decrease of L-lactate formation and pyruvate consumption in VWB-1.

**Figure 5:**
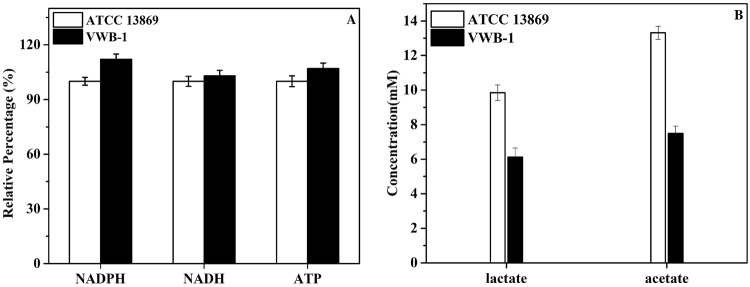
Relative level variation of intracellular NADPH, NADH and ATP (**A**) and the concentration of lactate and acetate comparison between *C. glutamicum* VWB-1 and ATCC 13869 (**B**).

In *C. glutamicum*, NADPH acts as a cofactor in the second step of L-valine biosynthesis in which 2-acetolactate was converted to 2,3-dihydroxyisovalerate by acetohydroxy acid isomeroreductase (AHAIR, *ilvC* encoded), it was reported to mainly generated through the oxidative pentose phosphate pathway (PPP)^20^. In the oxidative branch of pentose phosphate pathway, the 6-phosphogluconate dehydrogenase (encoded by *gnd*) catalyzes the formation of D-ribulose 5-phosphate from 6-phospho-D-gluconate, accompanied by the generation of NADPH, 6-phosphogluconate dehydrogenase was up-regulated in VWB-1, thus strengthening the PPP and promoting the supply of NADPH. As shown in Fig. 5(A), the NADPH level was detected increased in VWB-1. The products of pentose phosphate pathway are also important intermediate products for other biosynthesis, e.g., the D-ribose 5-phosphate is the substrate of PRPP (5-phospho-α-D-ribose 1-diphosphate) biosynthesis and PRPP is the pivotal intermediate of purine and pyrimidine *de novo* synthesis, another intermediate product D-erythrose 4-phosphate is the key substrate of chorismate biosynthesis.

The sufficient supply of biotin is also important for amino acids production. Biotin is a water soluble, heterocyclic cofactor for acetyl-CoA carboxylase, methylcrotonyl-CoA carboxylase, propionyl-CoA carboxylase and pyruvate carboxylase, those enzymes play essential roles in cell metabolism^21^. It could facilitate the transfer of CO2 during carboxylation, decarboxylation and transcarboxylation reactions involved in fatty acid and carbohydrate metabolism^22^. The two important enzymes involved in the biotin synthesis, the *bioA*-encoded 7,8-diaminopelargonic acid synthase and the *bioD*-encoded dethiobiotin synthase, separately catalyzes the first and second step of biotin biosynthesis, were both down-regulated in VWB-1, making it disabled in biotin biosynthesis. Hence, moderate amount of biotin should be added to the medium during the fermentation of VWB-1 to maintain a high cell viability and metabolic activity.

The fact that the glucose consumption rate of VWB-1 was lower than that of ATCC 13869 at the initial stage of fermentation could be explained by the proteomics results, for the uptake and utilize of carbon sources is crucial for cell growth and metabolism. It was noticed that the sugar metabolism transcriptional regulator SugR was up-regulated in VWB-1, not only in transcriptional level but also in translational level. The presence of SugR was reported to lead to the transcription suppression of a series of genes belonging to the PTS system, *ptsI*, *ptsH* and *ptsG*^23^, whose transcriptional level were all down-regulated (mentioned later). SugR is also the transcriptional repressor of enolase encoding gene *eno*, whose transcription and protein level were both decreased in VWB-1. Therefore, it might be possible to promote the carbon source uptake and utilization efficiency of VWB-1 by suppressing the expression of the *sugR* and reducing its transcription level inhibition effect on those above-mentioned genes.

### Proteins analysis in macroelement and metal homeostasis module, SOS and stress response

Proteins subjected to the macroelement and metal homeostasis module with varied expression intensity were identified. It was detected that the expression of *cysR* encoded Crp family protein CysR was increased in VWB-1. CysR is the dual transcriptional regulator of a subset of MbcR regulon genes including *fpr2*, *cysIXHDNYZ*^24^, it is also predicted to be the transcriptional activator of *ssuR* (https://coryneregnet.compbio.sdu.dk/v6e/CoryneRegNet/queryElement.php?gene=cg0012). The up-regulated sulfate adenyltransferase subunit 1 (*cysN* encoded) and a predicated nucleoside-diphosphate-sugar epimerase (*cg3375* encoded) were also under the transcriptional activation of CysR. However, the ATP-binding protein of the glutamate uptake system encoded by *gluA* was detected down-regulated, which might lead to the glutamate uptake deficiency of VWB-1^25^.

It is worth mentioning that the phosphate uptake regulator PhoU (*phoU* encoded), which plays a negative role in the regulation of phosphate uptake, was down-regulated in VWB-1, and another regulator PhoR (*phoR* encoded) was greatly up-regulated both in transcriptome and proteome. Wild-type *C. glutamicum* cells were reported to produce *phoRS* mRNA in phosphate limitation conditions, but not in Pi excess, showing the importance of PhoRS in Phosphate metabolism^26^.

The SOS and stress response is an inducible gene regulatory process that allows the cell to survive unexpected DNA damage^27,28^. The iron–sulfur cluster assembly system proteins SufB and SufD were up-regulated in VWB-1, the transcriptional level of another member of this system, the ATPase subunit SufC, was also increased. Iron-sulfur proteins are best known for their role in the oxidation-reduction reactions of electron transport, the SufBCD complex contributes to the assembly or repair of oxygen-labile iron-sulfur clusters under oxidative stress, and may facilitate iron uptake from extracellular iron chelators under iron limitation^29^. We also detected the up-regulation of the heat shock protein (HSP) encoded by *clpB* and the down-regulation of superoxide radicals degradation pathway catalase encoded by *katA*.

### Proteins weren’t clarified to any modules were also regulated

The expression level change of numerous proteins that have not yet been classified but still are important for the physiology of cells were observed. For instance, the expression variation of gene transcription and translation related proteins were detected. Two ribosomal proteins, the 30S ribosomal protein S6 (*rpsF* encoded) and the 50S ribosomal protein L10 (*rplJ* encoded) were up-regulated in VWB-1, both of them are the major components of the ribosome and play crucial role in cellular process of translation. In addition, the DNA repair and genetic recombination protein RecF (binds preferentially to single-stranded, linear DNA), the 23S ribosomal RNA methyltransferase TsnR and the translation elongation factor P (*efp* encoded) were all up-regulated in VWB-1, elongation factor P can stimulate efficient translation and peptide-bond synthesis on native or reconstituted 70S ribosomes *in vitro*, it can also increase the reactivity of ribosome as acceptors for peptidyl transferase by indirectly altering their affinity for aminoacyl-tRNA. Interestingly, the protein expression of another elongation factor Ts (EF-TS) was decreased, showing a discordant change with the translational level up-regulation of its coding gene *tsf*.

The transcription regulatory repertoire in *C. glutamicum* is a complex system including DNA-binding transcription regulators, response regulators of two-component systems and sigma factor subunits of RNA polymerase. Here we observed the protein expression level change of several transcriptional regulators in VWB-1, in addition to the aforementioned SugR, ArgR, Cg0156, PhoR and two elongation factors, elongation factor P and EF-Ts, we also observed the down-regulated protein expression of a TetR family transcriptional regulator (*cg0454* encoded) and a putative RNA polymerase sigma factor (encoded by *pvdS2*), the expression of an Zn-ribbon protein encoded by *cg2456* was up-regulated in VWB-1. Another up-regulated regulator of RNase E, the ribonuclease activity regulator protein RraA encoded by *menG*, can inhibit the catalytic activity of RNase E by interacting with it, thus increasing the half-life and abundance of RNAs.

In *C. glutamicum*, l-proline can be catabolized by the action of two enzymatic activities, in the two steps (both of which are catalyzed by a bifunctional dehydrogenase), proline is converted to l-glutamate, which can be further degraded to 2-oxoglutarate, an intermediate of the TCA cycle, and l-glutamate is also the amino donor of l-valine biosynthesis. The bifunctional proline dehydrogenase encoded by *putA* was significantly up-regulated in VWB-1. Expression level of another two important proteins involved in the degradation of glycogen, the glycogen phosphorylase encoded by *glgP1* and the 4-alpha-glucanotransferase encoded by *malQ*, were changed in VWB-1. Glycogen is the primary carbon and energy storage compound and may play a role in the long-term survival of the cell^30^. The up-regulation of the 4-alpha-glucanotransferase enhanced the cell ability of maltose utilization and gained more β-D-glucose 6-phosphate, which can afterwards enter the glycolysis pathway^31^.

### Transcriptome analysis biosynthetic pathway of l-valine was strengthened in *C. glutamicum* VWB-1

The transcriptional level regulation of the genes involved in the biosynthesis of l-valine and its branched metabolic pathway makes VWB-1 an ideal cell factory for l-valine production.

**Figure 6:**
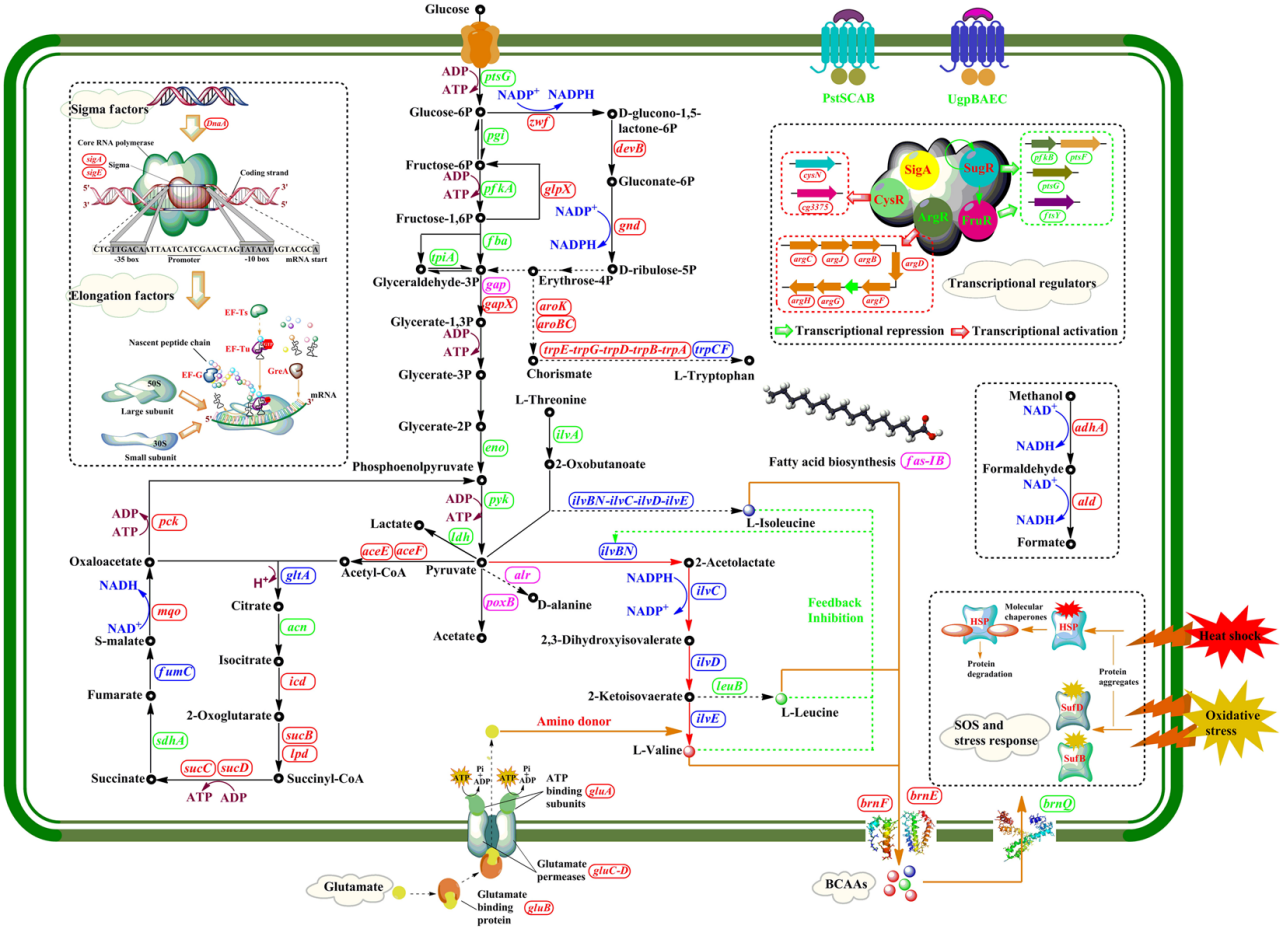
Overview of the transcriptional and translational regulation in L-valine production strain *C. glutamicum* VWB-1. Red box repesents the gene was up-regulated in transcriptional level; Green box represents the gene was down-regulated in transcriptional level; blue box represents the gene was up-regulated both in transcriptional and translational level; rose red box represents the gene was down-regulated both in transcriptional and translational level. Figure 6 was drawn by Hailing Zhang, the first author of this article.

As shown in Fig. 6, the pathway of l-valine biosynthesis in *C. glutamicum* is a four-step enzyme catalytic reaction that shares all its procedures with the parallel pathway of isoleucine biosynthesis^17,32^. These entwined pathways are part of the superpathway of branched amino acid biosynthesis, generating not only isoleucine and valine, but also leucine. The acetohydroxy acid synthase (AHAS) encoded by *ilvBN* catalyzes the first committed step in the biosynthesis of l-valine, l-leucine, and l-isoleucine. In this step, AHAS catalyzes the reaction of two molecules of pyruvate to form 2-acetolactate, it can also transfer the acetaldehyde from pyruvate to 2-oxobutanoate, forming 2-ethyl-2-hydroxy-3-oxobutanoate. Genes *ilvB* and *ilvN* encodes the catalytic subunit and the small, regulatory subunit of AHAS, respectively, they were separately up-regulated by 28.6 and 113.9 fold in strain VWB-1, which directs the carbon flow from pyruvate to L-valine biosynthetic pathway. Protein exercising the catalytic function in the second step of l-valine biosynthesis is acetohydroxy acid isomeroreductase (AHAIR) encoded by *ilvC*, which catalyzes the reaction of 2-acetolactate into 2,3-dihydroxy-3-methylbutanoate, employing coenzyme NADPH as the reductant and hydride donor, and the transcriptional level of *ilvC* was up-regulated by 15.4 fold in VWB-1. Following is the catalytic reaction of 2,3-dihydroxy-3-methylbutanoate into 3-methyl-2-oxobutanoate, this step is catalyzed by the enzyme 2,3-dihydroxy acid hydrolyase (DHAD), and the transcription of *ilvD*, the encoding gene of DHAD, was up-regulated by 2.8 fold. The *ilvE* gene of the last step was up-regulated by 5.9 fold, it encodes the branched-chain amino acid aminotransferase (TA) that transfers the amino group to 3-methyl-2-oxobutanoate, employing l-glutamate as the amino donor and generating the final product L-valine. Besides, several point mutations were found in the AHAS of VWB-1 (Figure S2A), and flask fermentation of the recombinant strains harboring the *ilvBN*^V^ *and ilvBN*^A^ separately cloned from VWB-1 and ATCC 13869 indicated that the AHAS of VWB-1 was more capable in L-valine producing (Figure S2B). The overall transcriptional up-regulation of the five key genes involved in the biosynthesis of L-valine above lead the maximum carbon metabolic flux flowing to the final product, thus promoting the L-valine production capabiliity of VWB-1.

The competitive pathways of l-valine biosynthesis were weakened in VWB-1, one of them was the biosynthesis of L-isoleucine. The L-isoleucine synthesis approach is started by the conversion of L-threonine into (2Z)-a-aminobut-2-enoate under the catalysis of the *ilvA* encoded threonine dehydratase (TD), and TD then catalyzes the following two spontaneous reactions to form 2-oxobutanoate as well, which is also a substrate of AHAS. It can be observed that in VWB-1, the transcriptional level of *ilvA* was down-regulated by 1.9-fold, showing a decrease in its L-isoleucine biosynthesis capability, thus reducing the branch substrate competition of AHAS and other key enzymes, and still reducing the purification and separation cost, this can be testified from the amino acids composition analysis of fermentation broth (Fig. 1A). There was also unfavorable transcriptional regulation existed in VWB-1, the transcriptional level of the alanine aminotransferase coding gene *alaT* was up-regulated by 5.1-fold, this might lead to the pyruvate competition from L-alanine synthesis. Nevertheless, it is worth mentioning that the production of L-alanine is decreased in VWB-1, probably due to the down-regulation of *alr*, which encodes the alanine racemase that turns D-alanine into l-alanine, alanine racemase was also down-regulated in proteomics. The same situation also happened in the 3-isopropylmalate dehydrogenase coding gene *leuB*, the down-regulation of *leuB* might be the cause that lead to the production decrease of l-leucine in VWB-1.

In addition to the DEGs involved in the biosynthesis of l-valine, a wide range of transcription profile changes were also detected among the genes participating in other amino acids metabolism in VWB-1. The transcriptional levels of the genes involved in aromatic family amino acids synthesis, e.g., the *trpA-E* of the L-tryptophan biosynthesis, *aroBC* and *aroK* that function in the synthesis of chorismate, which is an intermediate in the synthesis of three amino acids: l-phenylalanine, l-tyrosine and l-tryptophan^33^, they were all up-regulated by 2.6-6.5-fold, and *trpCF* encoded phosphoribosylanthranilate isomerase was up-regulated in translational level (mentioned above). In histidine metabolism, the transcription of *hisG* and *hisH* were promoted by 3.4–3.5 fold. Besides, it seemed that the degradation of L-aspartate was strengthened in VWB-1, because the mRNA level of the two genes participating this process, *aspA* and *ansA*, were all increased, showing that VWB-1 might be a candidate for the production of l-aspartate derived amino acids.

### Key precursors and cofactors in the biosynthetic pathway of l-valine were accumulated in the central metabolism of *C. glutamicum* VWB-1

**Figure 7:**
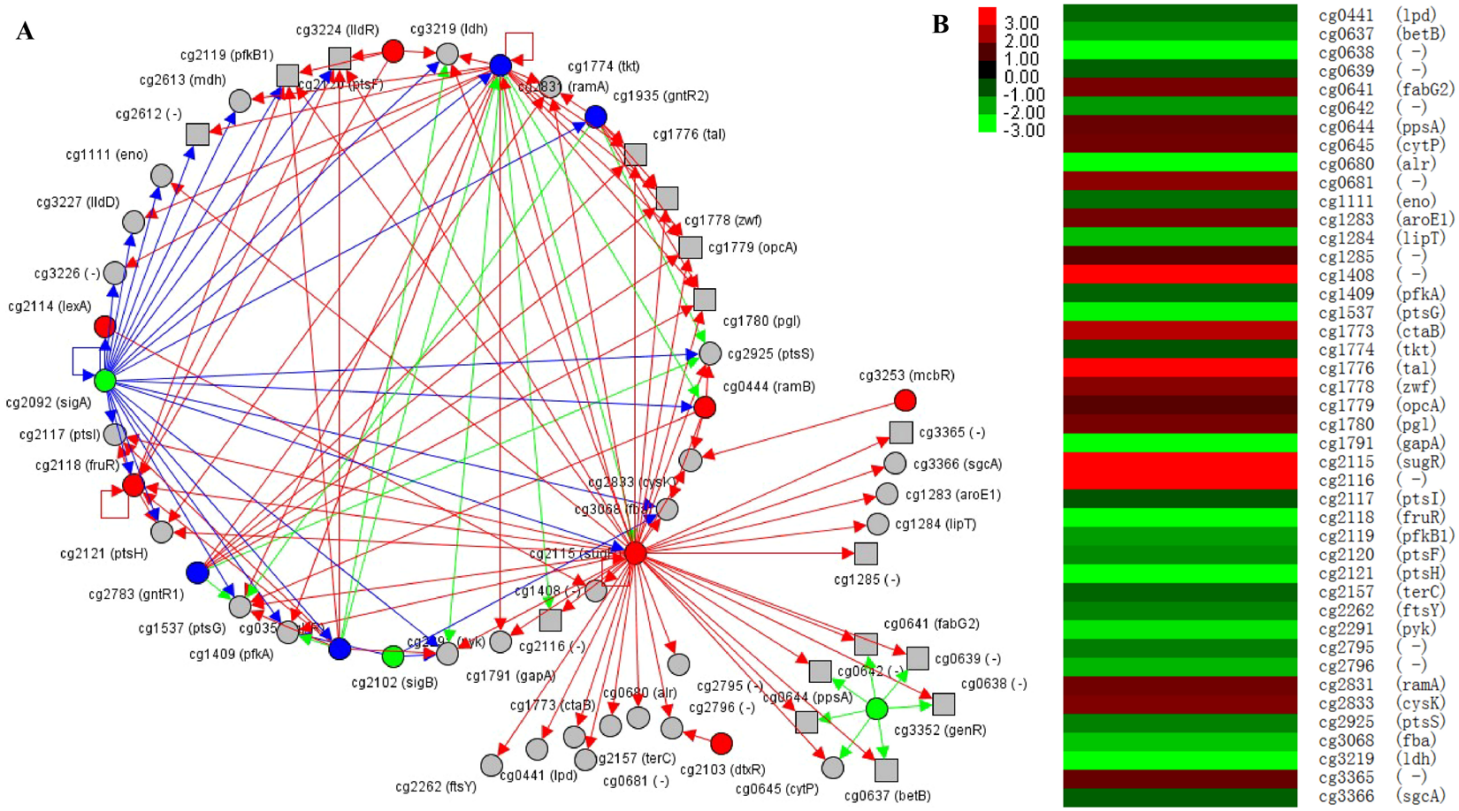
The regulatory network of sugar metabolism transcriptional regulator SugR in *C. glutamicum* VWB-1 (**A**) and the DEGs repressed by it (**B**). (**A**) was drawn on http://www.coryneregnet.de. Definition of the symbols are listed as follows, grey circle with black solid line, regulated target gene preceded by a transcription factor binding site; grey square with black solid line, regulated target gene that is part of an operon and not prededed by a transcription factor binding site; red circle with black solid line, repressor; green circle with black solid line, activator; blue circle with black solid line, dual regulator; organe circle with black solid line, homologenous to the selcted gene; grey circle with green dashed line, upstimulated gene; grey circle with red dashed line, downstimulated gene; red arrow, repressing regulatory interaction; green arrow, activating regulatory interaction.

The higher production of l-valine and improved transportation mean the higher requirements of substrate and energy supply. *C. glutamicum* is able to grow on various carbon and energy sources, such as sugars, sugar alcohols, and organic acids, with which glucose is the preferred carbon source^18,34^. The efficiency of glucose uptake and utilization directly influence the cell growth, metabolism and energy supply. As shown in Fig. 1(B,C), VWB-1 grows slower and its final cell density was lower than that of ATCC 13869, meanwhile, the glucose consumption rate of VWB-1 was slower than ATCC 13869 at the beginning of fermentation. Efforts have been made to discover the causes of this phenomenon by analyzing the transcriptional and translational changes of genes related to glucose utilization and carbon metabolism, the transcriptional change of a series transcriptional regulators were detected, including the transcriptional and translational up-regulated sugar metabolism transcriptional regulator SugR. Figure 7(A) shows the regulatory network of SugR in *C. glutamicum* ATCC 13032. RNAseq data indicates that the transcriptional level of *sugR* was up-regulated by 3.9-fold, the LuxR family transcriptional regulator RamA and RNA polymerase sigma factor SigA were up-regulated too, both them possess a positive regulation over *sugR*. In *C. glutamicum*, SugR was reported to function as a global repressor of genes of the PTS system, glycolysis, and fermentative lactate dehydrogenase^34–36^. The phosphoenolpyruvate-dependent phosphotransferase system (PTS) is involved in uptake and utilization of glucose, fructose and sucrose in *C. glutamicum*, and glucose-, fructose- and sucrose-specific enzymes of PTS are encoded by *ptsG*, *ptsF* and *ptsS*, respectively^37^. Purified SugR protein was demonstrated to repress the expression of *ptsG* and *ptsS* by binding to the upstream regions of the two genes *in vitro*, and the binding in front of the *fruR*-*pfkB1*-*ptsF* operon represses the transcription of the three genes^34^. In VWB-1, the transcriptional level of three PTS system genes, *ptsC*, *ptsS* and *ptsG* were separately down-regulated by −57.1, −30.7 and −2.8 under the negative regulation of SugR. Besides, the transcriptional regulator FruR and 1-phosphofructokinase PfkB1 were also transcription down-regulated, resulting a capability decline in carbon source and glucose utilization, perturbed growth on media containing glucose, which is consistent with the previous study^35^. Other down-regulated genes under the repression of SugR were *ftsY*, *pyk*, *fba*, etc., all of them were listed in Fig. 7(B) and play important roles in GTPase signal recognition, glycolysis and gluconeogenesis.

Glycolysis was first studied as the main pathway for the degradation of sugars, it is one of the major pathways of central metabolism, the other two being the pentose phosphate pathway and the TCA cycle^38^. In VWB-1, several genes involved in the glycolysis pathway, *cg0519*, *pck*, *glpX*, *glk* and *gapX*, etc., were observed up-regulated. It is worth mentioning that the transcriptional level of the gene *adhA*, which encodes a Zn-dependent alcohol dehydrogenase (ADH), was up-regulated by 66.7-fold in VWB-1, and the aldehyde dehydrogenase (ALDH) encoding gene *ald* was up-regulated by 24.0. The reactions catalyzed by ADH and ALDH were always accompanied by the generation of NADH, which showed a slightly increase in VWB-1 (Fig. 5A) and plays an important role in electron transport^39,40^, the together up-regulation of *adhA* and *ald* can help maintain the redox balance during fermentation.

In *C. glutamicum*, the pentose phosphate pathway is an alternative way of oxidizing glucose which is coupled to NADPH synthesis, whose level was measured raised in VWB-1 (Fig. 5A). NADPH was mainly generated in the preliminary oxidative portion of pentose phosphate pathway, in which β-D-glucose 6-phosphate is oxidized to D-ribulose 5-phosphate with the participation of three enzymes, the glucose-6-phosphate 1-dehydrogenase, the 6-phosphogluconolactonase and 6-phosphogluconate dehydrogenase separately encoded by *zwf*, *devB*, and *gnd*, transcriptional level of the three genes were all slightly increased in VWB-1, thus enhancing the regeneration of NADPH required for l-valine biosynthesis. Besides, the genes *rbsK1* and *glpX* involved in the pentose phosphate pathway were up-regulated by 4.7 and 4.0 respectively, contributing to the formation of NADPH, which is in favor of l-valine production.

The TCA pathway of VWB-1 was strengthened from the perspective of transcriptomics, ensuring the providing of precursor metabolites, energy and reducing power for cell metabolism, as it is a catabolic pathway of aerobic respiration, which generates both energy and reducing power and the first step in generating precursors for biosynthesis. The input reaction of acetyl-CoA into TCA cycle is catalyzed by the *gltA* encoded citrate synthase, whose transcriptional level was up-regulated by 4.4 in VWB-1. Transcriptional level of the aconitate hydratase encoding gene *acn* was down-regulated in the following step, which was restored by the up-regulation of the 2-methylcitrate synthase 2 coding gene *prpC2*. Isocitrate was then transformed into 2-oxoglutarate under the catalysis of the *icd* encoded isocitrate dehydrogenase, whose mRNA level was increased by 2.5 in VWB-1, this reaction is accompanied by the generation of NADPH, a significant cofactor in l-valine production, and still the 2-oxoglutarate generated in this reaction is an important biological compound that connects the intracellular carbon-nitrogen metabolism. The most obvious transcription change happened on the two succinyl-CoA synthetase subunit coding genes, the alpha subunit coding gene *sucD* and the beta subunit coding gene *sucC*, they were separately up-regulated by 53.2 and 8.3 in VWB-1, accelerating the mutual transformation between succinyl-CoA and succinate which is accompanied by the regeneration of ATP, which was measured increased in VWB-1 (Fig. 5A). Another transcription up-regulated genes in TCA cycle were the fumarate hydratase coding gene *fumC* (6.8 fold) and the malate:quinone oxidoreductase encoding *mqo* (3.9-fold), which could promote the interconversion between S-malate and fumarate and the following transformation of S-malate to oxaloacetate.

Pyruvate is the most important substrate in l-valine production process, many researchers have concentrated on increasing the accumulation and reducing the consumption of pyruvate to construct efficient l-valine production *C. glutamicum* strains^36,41–43^. In our results, transcriptional level of the pyruvate dehydrogenase gene *poxB* (−8.9) involved in the pyruvate fermentation to acetate pathway and the l-lactate dehydrogenase encoding gene *ldh* (−5.3) involved in the pyruvate fermentation to lactate pathway were both down-regulated, their translational levels were down-regulated, too. It can be observed in Fig. 5(B) that the concentration of acetate and lactate were both measured decreased in VWB-1, as the generation of lactate and acetate were both pyruvate consuming process, the down-regulation of *ldh* and *poxB* and the concentration decline of lactate and acetate both indirectly benefit the cell’s l-valine production capability by correlating to the accumulation of pyruvate. We also observed the down-regulation of two pyruvate kinase encoding genes, *pyk* (−2.7) and *cg3218* (−5.1), contributing to the consumption reduction of pyruvate.

### Transcriptional regulation of transportation capability of VWB-1

ATP-binding cassette transporters (ABC transporters) are transmembrane proteins implement certain biological process including translocation of various substances across membranes and non-transport-related processes, such as DNA repair and translation of RNA, accompanied by ATP binding and hydrolysis^44,45^.

The branched chain amino transportation capability of VWB-1 was enhanced. Transcriptome data showed that the branched chain amino acid transportation capability of VWB-1 has been greatly promoted. One important obstruction in the biosynthesis of l-valine is the feedback inhibition of l-valine to AHAS in the first step, along with the inhibition by l-isoleucine and l-leucine that together repress the catalytic activity of AHAS, and still the intracellular accumulation of these three amino acids perform a toxic effect on the cell^46,47^. In order to reduce these negative effects, l-valine should be transported to extracellular after being synthesized immediately. It has been confirmed by experiments that the protein BrnFE, composed of a small subunit BrnE and a large subunit BrnF, acts as the branched-chain amino acid efflux transporter that undertake the role of transferring the branched chain amino acids to extracellular, another protein BrnQ was reported to be the uptake transporter of all branched chain amino acids^48^. In *C. glutamicum*, BrnFE is the only carrier for export of l-isoleucine, l-leucine and l-valine and it can also export l-methionine^18,49,50^. On the one hand, the two genes *brnE* and *brnF* that encode the small and large subunit of BrnFE, respectively, were separately up-regulated by 4.0 and 25.4 (shown in Table S1), making VWB-1 better in branched chain amino acid transportation than ATCC 13869, on the other hand, the transcript level of *brnQ* that encodes the branched-chain amino acid uptake carrier was slightly decreased in VWB-1, indicating its branched-chain amino acid uptake capability declining. The improvement of branched chain amino acids transport capability, especially the transport of l-valine, could remove the intracellular l-valine toxicity and released its inhibitory effect on AHAS, thus be beneficial to l-valine production.

Glutamate was the amino donor of l-valine biosynthesis, the glutamate transport capability of VWB-1 showed an improvement according to the transcriptional up-regulation of the four genes forming the glutamate transport system, *gluB*, *gluC*, *gluD* and *gluA*, which separately encodes the substrate binding component, glutamate permeases (*gluC* and *gluD*) and ATP binding component. In the meantime, a series of iron complex transport system genes also showed an up-regulation between 2.4 and 10.6, such as three ATP-binding protein coding genes, *cg0928*, *cg0767* and *cg3129*, and two permease coding genes, *cg0926* and *cg0927*. These beneficial metabolic regulations might have a more profound effect on the metabolism of cells, as iron plays an important role in many oxidation-reduction reactions and can help promote the hydrogen peroxide stress response of *C. glutamicum*^51^.

The ATP-dependent phosphate uptake system composed of four components PstSCAB was weakened in VWB-1. As shown in Table S1., transcriptional level of the substrate-binding component encoded by *pstS* was down-regulated by −30.7 in VWB-1, the two permease proteins, separately encoded by *pstA* and *pstC*, were down-regulated by −71.3 and −57.1, respectively. The mRNA level of the ATP-binding protein encoding gene *ptsB* was down-regulated by −114.8 compared to that of ATCC 13869. The ATP-binding cassette (ABC) superfamily member PstSCAB is responsible for inorganic phosphate (Pi) uptake under Pi starvation conditions, for Pi is an essential component in cellular function since phosphorylation of nucleic acids, lipids, sugars, and proteins are important for gene regulation and signaling^52,53^. The down regulation of the Pst system indicates a declination of the cell Pi uptake capability and might result in the occurrence of phosphate starvation, as phosphorus is such an essential component that phosphorus metabolism is always closely intertwined with the energy and the central carbon metabolism, from which the precursor metabolites for the biosynthesis of amino acids are derived, this kind of down-regulation might affect the cell metabolism and synthesis of amino acids product of VWB-1 adversely. Another ABC transport system showing a unified transcriptional decrease is the UgpBAEC system, which is responsible for the transport of sn-glycerol 3-phosphate, a key precursor of membrane lipids and phospholipid synthesis. The four genes, *ugpA*, *upgE*, *ugpB* and *ugpC* all showed mRNA level decrease from −114.4 to −11.3. We also observed the transcriptional down-regulation of three permease coding genes, *cg1082*, *cg1647* and *cg3368*, and the ATP-binding gene *cg3367* of the antibiotic transport system, their mRNA level all showed a decrease between −13.9 and −2.1.

### Transcriptional regulation of elongation factor, ribosomal protein, sigma factors coding genes

The transcriptional level comparison of the genes encoding the elongation factors between *C. glutamicum* VWB-1 and ATCC 13869 are listed in Table S1. Elongation factors are a set of proteins that act in mRNA translation process by promoting the polypeptide chain elongation from the formation of the first peptide bond to the last one in ribosome. EF-Ts interacts with aminoacyl-tRNA-bound EF-Tu and regulates its affinity for GTP and aa-tRNA ligands, and EF-G can facilitate the translocation of the ribosome by one codon along the mRNA molecule^54^. As shown in Table S1, transcriptional levels of the genes encoding for elongation factors were all raised in VWB-1, meanwhile, a wide range of up-regulation was observed among the genes coding for both the 30S and 50S subunits of the ribosomes in VWB-1. The process of l-valine biosynthesis was accompanied by the synthesis of a series of proteins act as catalyzers or transporters, we have mentioned above that the transcriptional level of most of those genes were raised in VWB-1. However, the vast amount of protein translation and modification is a rather complex process which needs the active participation of elongation factors and ribosomal proteins, the up-regulation the elongation factors and ribosomal proteins coding genes is precisely the embodiment of this demand.

Sigma factors are proteins needed only for initiation of RNA synthesis, they act as mediators of sequence-specific transcription by bind to RNA polymerases and orchestrate transcription initiation, such that a slightly change of sigma factor can affect the transcription of all housekeeping genes or a set of genes the cell wishes to express in response to some external stimulus such as stress^55^. As shown in Table S1, the seven sigma factors detected in *C. glutamicum* ATCC 13032 are listed as SigA-E, SigH and SigM. Transcriptional level of *sigA* was increased by 3.9-fold in VWB-1, SigA is the transcriptional activator of more than 250 genes in *C. glutamicum*, most of them showed an increase in mRNA level according to the transcriptome sequencing results. The transcriptional level of SigE was also up-regulated, it binds with the catalytic core of RNA polymerase to produce the holoenzyme, and directs bacterial core RNA polymerase to specific promoter elements to initiate transcription. The rRNA guanine-N1-methyltransferase coding gene *rrmA* was up-regulated by 1.9 under its activation in VWB-1. On the contrary, transcription decrease was observed both in the extracytoplasmic function sigma factor encoding gene *sigD* and the ECF superfamily RNA polymerase sigma-70 factor coding gene *sigM*.

### Key genes involved in cell division and cell wall synthesis were transcriptional regulated in VWB-1

As shown in Table S1, the transcription level of *dnaA* that encodes the chromosomal replication initiator protein was up-regulated by 3.6 in VWB-1, DnaA could bind to the *dnaA*-box as an ATP-bound complex at the origin of replication during the initiation of chromosomal replication, and it can also affect transcription of multiple genes including itself^56^. At the same time, transcriptional levels of the gene *ftsX*, coding for a cell division protein, *ftsE*, coding for a cell division ATP-binding protein, *ftsZ*, encoding a cell division GTPase, and *ftsQ*, coding for cell division septal protein, were separately up-regulated by 15.8, 5.7, 3.6 and 3.5, their together up-regulation guarantee the effective and normal cell proliferation, furthermore, it is a better solution to increase the total number of cells and extend the duration of stable period when a single cell’s productivity reaches a certain bottleneck, which would finally promote the glucose conversion rate and l-valine production. As shown in Fig. 1(C), the glucose consumption rate of VWB-1 surpassed that of ATCC 13869 after the sampling time, during which period the up-regulation *fts* genes were observed. That’s because the production of amino acids and the cell multiplication caused by the cell division were both large glucose consumers. Many researchers have tried to improve the biotechnological production of amino acids in *C. glutamicum* through the manipulation of cellular growth rate or the properties of the bacterial cell envelope, higher amino acids efflux capability could also be achieved through the structure modification of cell wall^57,58^. mRNA levels of the cell division protein FtsI coding gene *ftsI*, the chromosome partitioning involved ATPase coding gene *parA2*, the cell envelope biogenesis S-adenosylmethionine-dependent methyltransferase encoding gene *mraW*, and the ATP-dependent protease coding gene *clpX* were all slightly increased by approximately 2.2–2.9 in VWB-1. However, the bacterial cell division membrane protein coding gene *ftsW* and the putative replicative DNA helicase coding gene *dnaB* were decreased moderately by −4.6 and −3.6.

## Discussion

Favorable progresses have been made in employing proteomics or transcriptomics to elucidate the underlying metabolic regulation mechanisms of *C. glutamicum* under different environmental conditions or stimulus^59–62^. In this study, these two advanced bioinformatics analysis methods were combined organically to reveal the l-valine biosynthesis mechanism of the high production strain VWB-1.

We found that the key genes involved in l-valine biosynthesis, *ilvBN*, *ilvC*, *ilvD* and *ilvE* were all transcriptional up-regulated, and the proteins encoded by *ilvB*, *ilvD* and *ilvE* all showed significant up-regulation in their protein level. Other researchers have reported their efforts in promoting the l-valine production by co-expression of these genes and all of them have made great achievements^5,16^. At the same time, the negative regulation of several key genes from the competitive pathway of l-valine synthesis were observed, such as the down-regulated *ilvA* from the l-isoleucine pathway, the down-regulation of *alr* from the l-alanine branch and the down-regulated *leuB* from the l-leucine branch. These regulations not only weakened the substrate competition effect but also reduced the generation of heteroacids, ensuring the high productivity and purity of l-valine, this could be verified by the amino acids composition analysis through HPLC. The two-component transporter BrnFE is the export system for branched-chain amino acids l-leucine, l-isoleucine and l-valine in *C. glutamicum*, the importance of the BCAA transportation system was also mentioned in many researches^7,18^. In our study, the *brnFE* operon that encodes the BrnFE was transcriptional up-regulated, thus strengthening the BCAA transportation capability of VWB-1, reducing feedback inhibition of these three BCAAs to AHAS, the cost of downstream separation and purification would be decreased as well. Genes involved in substrate generation, energy supply and cofactor synthesis were also positive regulated in VWB-1. NADPH was reported to be crucial in industrial amino acids production^19,63^, the NADPH and ATP generation capability of VWB-1 were strengthened through the up-regulation of the genes involved in phosphate pentose pathway and TCA pathway. All the favorable regulations mentioned above contributed to the fact that VWB-1 being able to yield larger production of l-valine under a stable growth state.

The combination of transcriptomics and proteomics analysis overcame the lacking of enough persuasion when only one technique was employed. We had demonstrated previous research achievements speculated or verified by other researchers, for instance, the transcriptional regulator SugR was responsible for the transcriptional inhibition of several sugar-related genes in the cell, which lead to low cell sugar uptake rate and hypometabolism^34,64^, this can also explain the phenomenon that the growth and glucose consumption rate of VWB-1 was lower than that of ATCC 13869 at the beginning of fermentation. However, according to our observation and analysis of the experimental results, we speculated that this kind of hypometabolism might be beneficial to the production of l-valine. Due to the cell’s low growth rate and metabolism activities, higher proportion of substrate in the culture medium was used to produce l-valine rather than cell growth, which was in consistent with the aim of industrial production, the down-regulation of pyruvate consumption pathways can also demonstrate this viewpoint. We have also found the up-regulation of factors involved in protein synthesis. For instance, the up-regulation of ribosome elongation factor EF-G was observed in VWB-1, EF-G was significantly up-regulated in an l-isoleucine producing *C. glutamicum* strain, and elongation factors EF-G and EF-Ts were reported to promote the synthesis of l-isoleucine in *C. glutamicum* in another research^65^, many prokaryotes have two copies of the gene encoding EF-Tu, showing the importance of their existence^66^. Meanwhile, a large percent of ribosomal protein coding genes were up-regulated, thus ensured the effective synthesis of those enzymes required in l-valine production and transportation. It could also be demonstrated in this study that VWB-1 was more than an l-valine producing strain based on its excellent characteristics, which could be genetically modified to produce those important products employing pyruvate as a substrate, D-alanine and l-isoleucine, for example.

With the extensive application of various high-throughput sequencing techniques, a mass of data can be acquired from the genomics, transcriptomics, proteomics and metabonomics, the valuable information exploration capability was strengthened with the gradually improved bioinformatics methods, and system biology emerged under this background. System biology is also known as the metabolic engineering of the omics era^67–69^, it is the study of the composition of all genomics, transcriptomics, proteomics and metabonomics in the biological system and the interrelationship between these subjects under specific conditions, which can reveal the global bioprocesses and their regulation mechanisms, thus creating the possibility to illuminate the regularity of cell physiology activity on the whole. The biosynthesis of l-valine was a rather complex system, the l-valine productivity of a *C. glutamicum* strain might be affected by several intracellular and extracellular factors, thus the blindly employment of strain breeding strategies was always time and money cost. When certain bottleneck appeared in the strain optimization process, the combination employment of two or more bioinformatics analysis could deeply explore and explain the unique physiological metabolism mechanism underlying the surface, and those reasonable and efficient manipulation methods could be implemented to make the industrial strains becoming more efficient workhorses in the future.

## Methods

### Bacterial strains and growth

*C. glutamicum* strain VWB-1 and ATCC 13869 were cultured in a modified LBGB (LBG added with 1.85% brain heart infusion) medium. The L-valine producing mutant *C. glutamicum* strain (VWB-1) was derived through a screening and selection process. Briefly, bacteria were originally isolated from soil and treated with the mutagen diethylsulfate. VWB-1 was selected on agar plates containing L-valine structural analogues, such as α-aminobutyric acid and sulfaguanidine, and it was predicted to be derived from *C. glutamicum* ATCC 13869 based on the sequence similarity of its 16 S rDNA. For fed-batch fermentation, the VWB-1 and ATCC 13869 strains were cultivated in a fermentation medium which contains 120 g·liter^−1^ glucose, 1 g·liter^−1^ KH_2_PO_4_, 0.4 g·liter^−1^ MgSO_4_, 10 mg·liter^−1^ FeSO_4_, 10 mg·liter^−1^ MnSO_4_, 40 g·liter^−1^ (NH_4_)_2_SO_4_, 10 ml·liter^−1^ soybean meal hydrolysate, 0.7 g·liter^−1^ DL-methionine, 50 μg·liter^−1^ biotin, 300 μg·liter^−1^ vitamin B_1_, 0.25 g·liter^−1^ L-isoleucine. Before the fermenter system cultivation, pre-cultures were grown in seed culture medium in shaking flasks which contains 30 g·liter^−1^ glucose, 1 g·liter^−1^ KH_2_PO_4_, 0.4 g·liter^−1^ MgSO_4_, 10 mg·liter^−1^ FeSO_4_, 10 mg·liter^−1^ MnSO_4_, 2 g·liter^−1^ (NH_4_)_2_SO_4_, 60 ml·liter^−1^ soybean meal hydrolysate, 0.1 g·liter^−1^ DL-methionine, 50 μg·liter^−1^ biotin, 200 μg·liter^−1^ vitamin B_1_. During the 5 L fed-batch fermentation process, the culture temperature was set as 31.5 °C, the dissolved oxygen level was maintained at 30%, pure oxygen was fed into the fermentator if necessary, 80% glucose aqueous solution was added into the fermentation system when the glucose concentration was below 20 g liter^−1^, and the pH was automatically adjusted to 7.2 with 50% ammonia, which was also used as the supplement of nitrogen source. At the OD_562nm_ of 40, 20 ml *C. glutamicum* cell broth of middle exponential growth phase was harvested for proteome analysis and 1 ml was harvested for transcriptome analysis. The fermentation process was continued till the end of the stationary phase, and 1 ml final fermentation broth was harvested for amino acid composition analysis with the employment of HPLC Agilent 1260 (Agilent, USA). For organic acids analysis, firstly, fermentation broth sample was centrifuged and washed twice with PBS buffer, cell disruption was carried out by freezing-ultrasonic disruption method, trichloroacetic acid was added to remove the residual protein. Secondly, the organic acids concentration measurement was conducted by Agilent 1200 chromatography system, Waters XSelct HSS T3 C18 (250 mm*1.6 mm, USA) was selected as the chromatography column, preparation of mobile phase was prepared as previous research^70^. Flow speed was set as 0.5 mL/min, detection of wave length was set as 210 nm. Finally, intracellular concentration of organic acids was calculated the same as amino acids calculation method. NADPH, NADH and ATP level were measured as previous research^71^. The NADPH, NADH and ATP level of ATCC 13869 were defined as 100%, and the relative percentage of NADPH, NADH and ATP level in VWB-1 were calculated by dividing the correspondent level of ATCC 13869 with its own level.

### Two-dimensional electrophoresis and results analysis

#### Protein sample preparation

Cell disruption and membrane preparation were carried out based on the improvement of the previous research^72^. Bacterial samples were washed twice with PBS buffer, and then washed twice with ddH_2_O to remove the remaining buffer. After being dealt with liquid nitrogen grinding method, 1.5 ml lysis buffer was added into the sample, sonicate the mixture in ice bath for 20 s (0.5 s interval) in the presence of proteinase inhibitor PMSF, repeat this step five times. Centrifuge the mixture for 30 min at 4 °C with a speed of 12000 rpm, remove the supernatant and add 1.5 ml TCA-acetone solution (10%), lay the mixture at −20 °C for 12 hours. Centrifuge the mixture for 30 min at 4 °C and 12000 rpm again and discard the supernatant, add a certain amount of 90% acetone, repeat once. Add a suitable amount of lysis buffer, sonicate the mixture in ice bath for 20 s (0.5 s interval). Centrifuge the mixture at 4 °C and 12000 rpm for 30 min, measure the protein concentration of the supernatant by Bradford method^73^ and place under −80 °C. The lysis buffer was composed of 8 M urea (BBI, Canada), 2 M thiourea (TCRY, China), 2% (w:v) CHAPS (Sigma, USA), 2% (w:v) PH3-10 ampholyte (Bio-Rad, USA), 1% DTT (Sigma, USA) and 1 mM PMSF (Sigma, USA).

#### First dimensional isoelectric focusing (IEF)

For isoelectric focusing, 24-cm precast IPG strips, pI 4–7, and IPGphor isoelectric focusing unit (Amersham Biosiences, Freiburg, Germany) were used as described previously^74^. Take out the prefabricated IPG strips (24-cm, pI 4 to 7, Bio-Rad, USA) from the refrigerator and swell for 12 hours using the swelling buffer which contains 8 M urea, 2 M thiourea, 0.5% CHAPS, 0.52% ampholyte, 0.02% bromophenol blue (Bio-Rad, USA) and 1% DTT. Put the swelled IPG strip into the focusing groove and add enough mineral oil to submerge the sample loading cup, the loading quantity of protein sample was 800 μg, which was first diluted to 170 μl with swelling buffer. Close the lid in accordance with the positive and negative poles, for improved sample entry, low voltage (0–500 V) was conducted at the beginning of IEF procedure and the sample was focused for 50, 000 V·h. After the IEF program, balancing the IPG strip immediately and prepare for the second dimension SDS-PAGE electrophoresis.

#### Second dimensional SDS-PAGE electrophoresis

Two pieces of 12% polyacrylamide gels, balancing buffer I and balancing II were prepared, balance buffer I was composed of 50 mM Tris-HCl (pH 6.8), 6 M urea, 30% glycerol, 2% SDS, 2% DTT and 0.02% bromophenol blue, balance buffer II was composed of 50 mM Tris-HCl (pH 6.8), 6 M urea, 30% glycerol, 2% SDS, 2.5% iodoacetamide (Sigma) and 0.02% bromophenol blue. Put the swelled strip into the sample hydration tray filled with balance buffer I and shake horizontally for 15 minutes, then transfer the strip into balance buffer II and shake horizontally for another 15 minutes. After the second balancing step, take the IPG strip out of the sample hydration tray and put it into the upper side of the polyacrylamide gels, spot the marker into one side of the gel which was on the same side with the pH 4 strip end, 5% agarose solution was used to seal up the strip and marker, make sure there was no bubble existed and fix the gel sheet in the electrophoresis chamber. Fill the chamber with electrophoresis buffer and turn on the power switch, the start current was set as 15 mA for 15 minutes, the following step was performed under the 250 v constant voltage until bromophenol blue arrived the bottom, pry up the two layers of the glass and remove the gel gently at the end. Put the gel into a leveling tray containing staining solution, move the leveling tray onto a horizontal shaker incubator and shake overnight. The staining solution is composed of 10% (NH_4_)_2_SO_4_, 10% H_3_PO_4_, 0.12% coomassie brilliant blue G250 and 20% CH_3_OH^75^. After staining, transfer the gel into destaining solution (3% acetic acid) and shaking horizontally overnight.

#### Gel scanning and protein identification

Image analysis was started from the gel scanning under 300DPI, these DEPs were detected using the PDQuest 8.0.1 software (Bio-Rad, USA) according to their spot gray. The coomassie-stained gels were aligned using the PDQuest 8.0.1 software. All samples were separated at least twice by 2-D polyacrylamide gel electrophoresis (PAGE) to minimize irregularities (technical replicates), and biological replicates were performed to validate the results. Proteins were regarded as regulated if (i) the corresponding ratios referring to the relative gray of the spots were changed more than twofold and if (ii) this regulation pattern was found in all biological and technical replicates^76^.

### MALDI-TOF-MS

Protein spots of interest were excised from the gels for matrix-assisted laser desorption ionization time-of-flight (MALDI/TOF) mass spectra analysis.

#### Sample preparation

Gels were cut into 1 cubic millimeters blocks and washed with ddH_2_O in centrifuge tubes. 100 μl pH 8.0 50% (v:v) ACN (Fisher, USA) plus 25 mM NH_4_HCO_3_ (Fisher, USA) were used to destain the gels for 15 minutes, repeat 3 times until all the color faded. Immerse the gel blocks into 30 μl 100% ACN for dehydration until they turn white, drain the water out at room temperature. Tryptic in-gel digestion was conducted by the addition of 8 μl trypsin (0.005 mg ml^−1^) (Promega, USA) at 37 °C for 16 h.

#### Mass spectrometer operating procedures

Clean the sample plate, draw 0.3 μl tryptic digestion solution from the tube and mix with 0.3 μl matrix CHCA (Fluka, USA) on the sample plate, dry in the air. Open the 4000 series software (ABI, USA) and put the sample plate into the mass spectrometer. Create a new spotset, open the acquisition method and processing mode of the calibrating instrument, calibrate the instrument. Open the acquisition method and processing mode of the unknown sample, employ the 4200 first class laser intensity, 700 to 4000 Da molecular weight range detection and positive ion reflector mode to conduct the mass spectrometry analysis. All database searches were performed using the GPS Explorer^TM^ software, version 3.5 (ABI, USA). Create a new project under the gel mode, choose a spotset in a new retrieve and select MS, then compare the peptide fragment mass analysis results with the NCBI (nr) database (http://www.ncbi.nlm.nih.gov/) and the Swissprot database (http://www.expasy.org/proteomics/). For mass spectrometry analysis, the search parameters were set as follows, the precursor tolerance between the peptide fragments was confined to ±0.1 Da, one missed cleavage was permitted, carbamidomethyl-Cys was selected for protein fixed modifications and oxidation variable modifications were allowed, for MS/MS analysis, the precursor tolerance between the peptide fragments was confined to ±0.5 Da and the laser intensity was set as 4600. Identifications can be considered successful if Protein score C.I.% (first stage mass spectrometry, MS) or total Ion Score C.I.% (second stage mass spectrometry, MS/MS) was greater than or equal to 95.

### mRNA sequencing and transcriptome analysis

For transcriptome analyses, 500 μl *C. glutamicum* fermentation broth was centrifuged at 4 °C with a speed of 12000 rpm, 5 μl lysozeme (100 mg·ml^−1^) was then added into the resuspended tube and incubated at 37 °C for 30 minutes. After cell disruption, total cell RNA was extracted using the Simply P Total RNA Extraction Kit (BioFlux, Bioer Technoligy Co., Ltd.). Further processing with DNase on-column treatment was carried out according to the manufacturer’s instructions and rRNA was also removed to reduce sequencing interference. Fragmentation buffer was added for interrupting mRNA to short fragments, random hexamer-primer were used to synthesize the first-strand cDNA taking these short fragments as templates. Then the second-strand cDNA was synthesized in a reaction system composed of buffer, dNTPs, RNase H and DNA polymerase I. These short fragments were purified with QiaQuick PCR extraction kit and resolved with EB buffer for end reparation and poly(A) addition. After that, these short fragments were connected with sequencing adaptors. For amplification with PCR, suitable fragments were selected as templates according to the result of agarose gel electrophoresis. At last, the constructed library was sequenced using Illumina HiSeq™ 2000. Images generated by sequencers were converted by base calling into nucleotide sequences, which were called raw data or raw reads and were stored in FASTQ format. FASTQ files are text files that store both reads sequences and their corresponding quality scores. Dirty raw reads which contain adapters, unknown (larger than 10%) or low quality bases (more than half of the bases’ qualities were less than 5) will negatively affect following bioinformatics analysis, therefore, these data were discarded and clean reads were obtained for granted.

These filtered clean reads were the basis of the following analysis, they were separately mapped to the *C. glutamicum* ATCC 13032 genome (NCBI accession no. NC_006859. 1) with the employment of the Bowtie aligner (http://bowtie-bio.sourceforge.net/index.shtml). The first step was the sequencing quality assessment including alignment statistics using SOAP2^77^, statistics of rRNA, randomness sequencing assessment and distribution or reads in reference ATCC 13032 genome^78^. Followed was the gene expression annotation composed of gene coverage ratio calculation, the calculation of unigene expression using RPKM method (Reads per kb per million reads)^79^. Third was the gene expression difference analysis covering from the differentially expressed genes (DEGs) filtering analysis^80^, expression pattern and gene ontology functional enrichment analysis for DEGs, GO functional (http://www.geneontology.org/) and KEGG pathway analyses (http://www.kegg.jp/). During DEGs filtration analysis, the number of hypothesis tests were thousands, thus making the customary suitable p-value for individual test not enough to guarantee low false discovery rate, we have to do multiple testing correction to decrease the p-value for each individual hypothesis testing to guarantee the low false discovery rate in whole. Finally, we used a statistical method, the false discovery rate (FDR)^81^, to correct for p-value in multiple hypothesis, and we chose those DEGs with FDR ≤0.001 and the RPKM ratio lager than 2 as credible. We then performed cluster analysis of gene expression patterns with cluster software^82^ and Java Treeview software^83^ to classify genes with similar expression patterns, which usually mean functional correlation. We also conducted the international standardized gene functional classification in Gene Ontology (GO) system. The three GO ontologies: molecular function, cellular component and biological process together offer a dynamic-updated controlled vocabulary and a strictly defined concept to comprehensively describe properties of genes and their products in *C. glutamicum*^84^. Since different genes usually cooperate with each other to exercise their biological functions, pathway based analysis helps to further understand genes biological functions. In pathway enrichment analysis, we identified significantly enriched metabolic pathways and signal transduction pathway in DEGs comparing with the whole *C. glutamicum* genome background based on the major public pathway-related database, KEGG^85^. Pathways with Q-value ≤ 0.05 are significantly enriched in DEGs, it is necessary to mention that Q-value is defined to be the FDR analogue of the p-value and the Q-value of an individual hypothesis test is the minimum FDR at which the test maybe called significant. For each comparison of interest, two independent experiments were performed.

### Statistical analysis

Each analysis was conducted three times and the acquired data were presented as the standard error of the mean. The differences between the means of the test were calculated by one-way analysis of variance according to the Tukey’s post hoc test. The statistical analysis of the data was carried out by SPSS 15.0.

## Electronic supplementary material

Supplementary Information
