# Supplementary material for: Understanding the high l-valine production in Corynebacterium glutamicum VWB-1 using transcriptomics and proteomics
Source: Sci Rep. 2018 Feb 26;8:3632. doi: 10.1038/s41598-018-21926-5 (PMC5827029; doi:10.1038/s41598-018-21926-5)
Supplement: Supplementary file 1 — Supplementary Information [file 41598_2018_21926_MOESM1_ESM.docx]

**Understanding the high l-valine production in *Corynebacterium glutamicum* VWB-1** **using transcriptomics and proteomics**

SUPPLEMENTARY INFORMATION

Hailing Zhang^1,2^, Yanyan Li^1^, Chenhui Wang^1^ and Xiaoyuan Wang^1,2,3^

^1^State Key Laboratory of Food Science and Technology, ^2^School of Biotechnology, and ^3^Synergetic Innovation Center of Food Safety and Nutrition, Jiangnan University, Wuxi 214122, China.

Corresponding author：

Prof. Xiaoyuan Wang

State Key Laboratory of Food Science and Technology

Jiangnan University

1800 Lihu Avenue

Wuxi 214000

China

Tel: +86-510-85329236

Fax: +86 510 85329236

E-mail: xwang@jiangnan.edu.cn

Figure S1. Original gel scan image of Figure 2(A) and Figure 2(B), Figure 2(A) and Figure 2(B) were cut from Figure S1 (A) and Figure S1(B), separately.


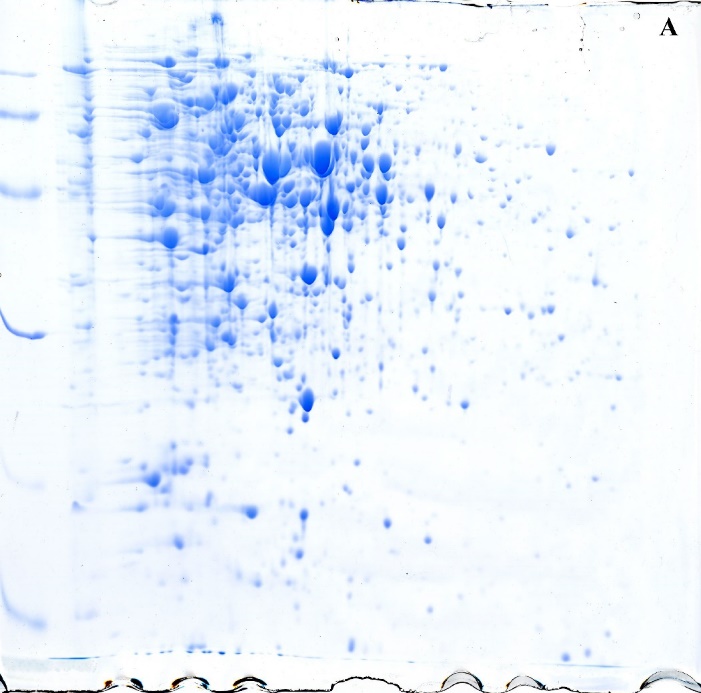


Figure S1(A). Original gel scan image of Figure 2(A)


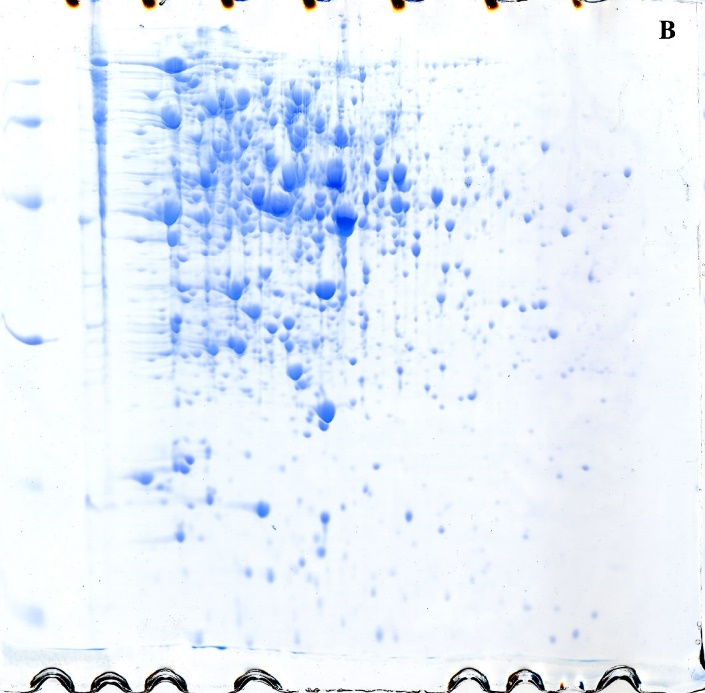


Figure S1(B). Original gel image of Figure 2(A)

Figure S2. Amino acids sequence alignment of IlvB between *C. glutamicum* VWB-1 and ATCC 13869 (A) and their contribution comparison in l-valine production. (a-b) represent the statistically significant differences (b>a).


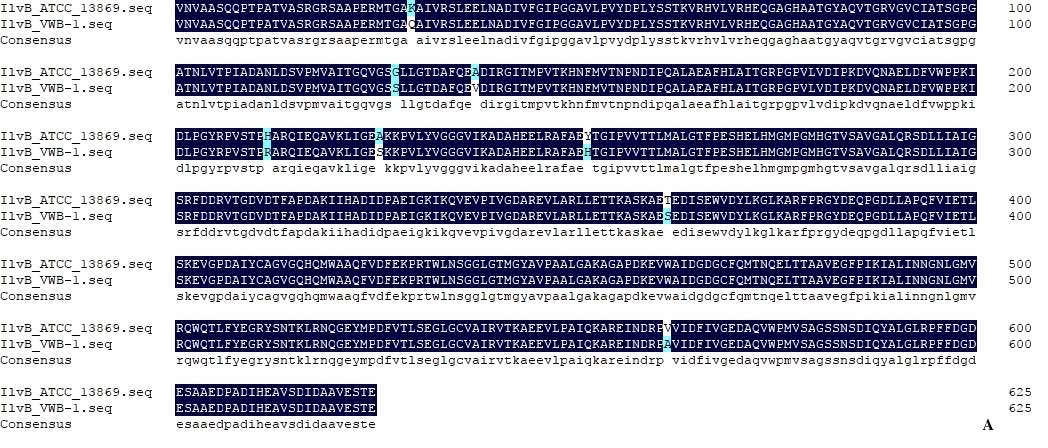


Figure S2 (A). Amino acids sequence alignment of IlvB between *C. glutamicum* VWB-1 and ATCC 13869


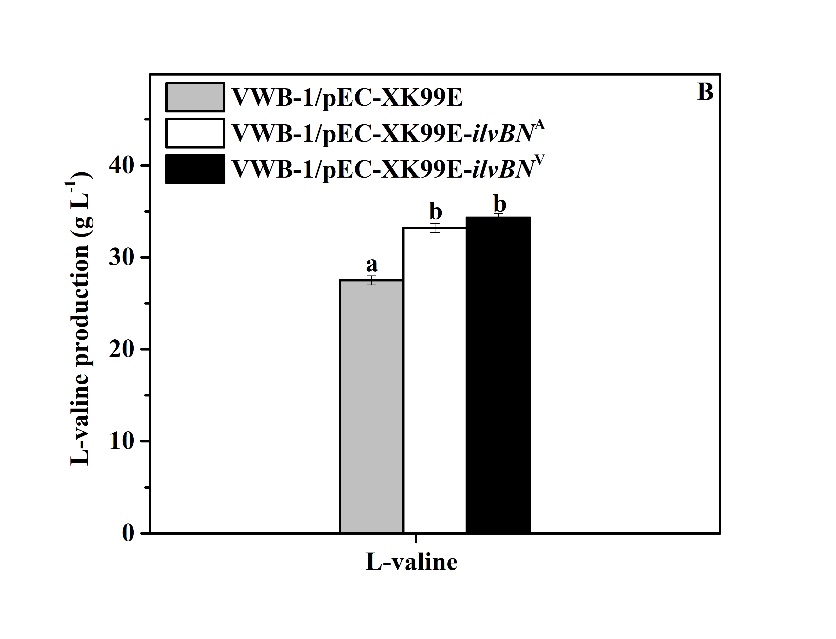


Figure S2 (B). l-valine production comparison between the recombinant *ilvBN* overexpression strains

Table S1. Gene transcriptional levels comparison between *C. glutamicum* VWB-1 and ATCC 13869

| Gene ID | Gene Name | Annotation | Regulation |
| --- | --- | --- | --- |
| **Amino acid biosynthesis** | | | |
| **Glutamate family** | | | |
| *cg2586* | *proA* | Gamma-glutamyl phosphate reductase | 6.0 |
| *cg2368* | *murC* | Probable UDP-N-acetylmuramate--alanine ligase protein | 3.5 |
| *cg0563* | *rplK* | 50s ribosomal protein L11 | 3.2 |
| *cg1493* | *ddl* | D-alanine-D-alanine ligase A | -9.2 |
| **Alanine, aspartate and glutamate metabolism** | | | |
| *cg1215* | *nadC* | Putative nicotinate-nucleotide pyrophosphorylase | 18.5 |
| *cg1816* | *pyrB* | Aspartate carbamoyltransferase catalytic chain | 9.8 |
| *cg2116* | *-* | Putative phosphofructokinase | 9.7 |
| *cg1814* | *carA* | Carbamoyl phosphate synthase small subunit | 8.9 |
| *cg0564* | *rplA* | 50s ribosomal protein L1 | 7.4 |
| *cg0129* | *putA* | Proline dehydrogenase | 5.3 |
| *cg3149* | *-* | Aminotransferases class-I | 5.1 |
| *cg2447* | *glnA2* | Glutamine synthetase 2 | 4.2 |
| *cg1697* | *aspA* | Aspartate ammonia-lyase (aspartase) | 3.3 |
| *cg2352* | *ansA* | L-asparaginase | 3.1 |
| *cg1586* | *argG* | Argininosuccinate synthase | 2.8 |
| *cg2399* | *glk* | Glucokinase, transcriptional regulator | 2.8 |
| *cg2876* | *purB* | Adenylosuccinate lyase | 2.5 |
| *cg1813* | *carB* | Putative carbamoyl-phosphate synthase subunit | 2.4 |
| *cg1588* | *argH* | Argininosuccinate lyase | 2.2 |
| *cg2429* | *glnA* | Glutamine synthetase i | 2.1 |
| *cg2410* | *ltsA* | Glutamine-dependent amidotransferase | 2.1 |
| *cg0067* | *gabD3* | Succinate-semialdehyde dehydrogenase (NADP^+^) | -3.9 |
| *cg0402* | *rmlCD* | DTDP-4-dehydrorhamnose 3,5-epimerase | -2.3 |
| **Glycine, serine and threonine metabolism** | | | |
| *cg0519* | *-* | Putative phosphoglycerate mutase | 8.0 |
| *cg0306* | *lysC* | Aspartokinase lysc alpha and beta subunits | 5.0 |
| *cg0307* | *asd* | Aspartate-semialdehyde dehydrogenase | 4.8 |
| *cg3375* | *-* | Predicted nucleoside-diphosphate-sugar epimerase | 4.6 |
| *cg2421* | *sucB* | Dihydrolipoamide succinyltransferase | 3.5 |
| *cg3364* | *trpA* | Tryptophan synthase alpha chain | 3.5 |
| *cg1451* | *serA* | Phosphoglycerate dehydrogenase | 2.6 |
| *cg3363* | *trpB* | Tryptophan synthase beta chain | 2.6 |
| *cg2587* | *-* | Phosphoglycerate dehydrogenase or related dehydrogenase | 2.5 |
| *cg2455* | *-* | Ribonuclease HI | 2.4 |
| *cg2845* | *pstC* | Permease component of phosphate transport | -57.1 |
| *cg2073* | *-* | Hypothetical protein | -6.9 |
| *cg1781* | *soxA'* | Sarcosine oxidase-fragment | -3.9 |
| *cg2890* | *-* | Putative amino acid processing enzyme | -3.6 |
| *cg0237* | *-* | Putative oxidoreductase | -3.6 |
| *cg1711* | *-* | Oxidoreductase | -3.3 |
| *cg1783* | *soxA* | Sarcosine oxidase-N-terminal fragment | -3.0 |
| *cg2852* | *-* | Putative aminomethyltransferase, GCVT homolog | -2.8 |
| *cg2987* | *dacB* | Penicillin-binding protein, D-Ala-D-Ala carboxypeptidase | -2.6 |
| *cg0344* | *fabG1* | 3-oxoacyl-(acyl-carrier protein) reductase | -2.6 |
| *cg2141* | *recA* | DNA recombination/repair | -2.0 |
| *cg1740* | *-* | Putative nucleoside-diphosphate-sugar epimerase | -2.0 |
| **Valine, leucine and isoleucine biosynthesis** | | | |
| *cg1436* | *ilvN* | Acetohydroxyacid synthase small subunit | 113.9 |
| *cg1435* | *ilvB* | Acetolactate synthase | 28.6 |
| *cg1437* | *ilvC* | Ketol-acid reductoisomerase | 15.4 |
| *cg2418* | *ilvE* | Branched-chain amino acid aminotransferase | 5.9 |
| *cg1487* | *leuC* | 3-Isopropylmalate dehydratase large subunit | 5.4 |
| *cg3149* | *-* | Aminotransferases class-I | 5.1 |
| *cg1488* | *leuD* | 3-Isopropylmalate dehydratase (small subunit) | 4.7 |
| *cg0303* | *leuA* | 2-Isopropylmalate synthase | 3.2 |
| *cg1432* | *ilvD* | Dihydroxy-acid dehydratase | 2.8 |
| *cg0601* | *rpsC* | 30s ribosomal protein s3 | 2.4 |
| *cg1684* | *tatC* | Sec-independent protein secretion pathway component | 2.4 |
| *cg3368* | *-* | ABC-transporter permease protein | -5.8 |
| *cg1082* | *-* | Putative membrane protein | -5.2 |
| *cg3381* | *-* | Sec-independent protein secretion pathway component | -3.6 |
| *cg0503* | *aroD* | Probable 3-dehydroquinate dehydratase | -2.1 |
| *cg1647* | *-* | Permease component of multidrug transport system | -2.1 |
| **Aromatic family** | | | |
| *cg1827* | *aroB* | Probable 3-dehydroquinate synthase protein | 6.5 |
| *cg1828* | *aroK* | Shikimate kinase i | 4.3 |
| *cg1829* | *aroC* | Putative chorismate synthase | 5.6 |
| *cg3359* | *trpE* | Anthranilate synthase component I | 5.9 |
| *cg3361* | *trpD* | Anthranilate phosphoribosyltransferase | 5.9 |
| *cg3362* | *trpCF* | Indole-3-glycerol-phosphate synthase | 3.4 |
| *cg3363* | *trpB* | Tryptophan synthase beta chain | 2.6 |
| *cg3364* | *trpA* | Tryptophan synthase alpha chain | 3.5 |
| *cg0503* | *aroD* | Probable 3-dehydroquinate dehydratase | -2.1 |
| **Histidine metabolism** | | | |
| *cg3096* | *ald* | Aldehyde dehydrogenase | 24.0 |
| *cg1354* | *rho* | Transcription termination factor Rho | 4.4 |
| *cg3070* | *-* | Spou rRNA methylase family protein | 4.2 |
| *cg3186* | *cmt2* | Trehalose corynomycolyl transferase | 3.9 |
| *cg3182* | *cop1* | Trehalose corynomycolyl transferase | 3.6 |
| *cg2300* | *hisH* | Imidazoleglycerol-phosphate synthase, amidotransferase | 3.5 |
| *cg1698* | *hisG* | ATP phosphoribosyltransferase | 3.4 |
| *cg0310* | *katA* | Catalase | 3.3 |
| *cg2883* | *-* | SAM-dependent methyltransferase | 2.6 |
| *cg2084* | *-* | Putative rna methyltransferase | 2.2 |
| *cg2824* | *-* | Sam-dependent methyltransferase | -14.5 |
| *cg2713* | *dhaS* | NADP-dependent aldehyde dehydrogenase | -10.0 |
| *cg1104* | *-* | Predicted esterase, membrane protein | -5.1 |
| *cg2953* | *xylC* | Benzaldehyde dehydrogenase | -2.8 |
| **Central carbon metabolism** | | | |
| **Glycolysis** | | | |
| *cg3107* | *adhA* | Zn-dependent alcohol dehydrogenase | 66.7 |
| *cg3096* | *ald* | Aldehyde dehydrogenase | 24.0 |
| *cg0519* | *-* | Putative phosphoglycerate mutase | 8.0 |
| *cg3169* | *pck* | Probable phosphoenolpyruvate carboxykinase protein | 4.9 |
| *cg1157* | *glpX* | GLPX-like protein | 4.0 |
| *cg2399* | *glk* | Glucokinase, transcriptional regulator | 2.8 |
| *cg1069* | *gapX* | Similar to glyceraldehyde-3-phosphate dehydrogenase | 2.6 |
| *cg2455* | *-* | Ribonuclease HI | 2.4 |
| *cg2091* | *ppgK* | Polyphosphate glucokinase | 2.1 |
| *cg2845* | *pstC* | Abc-type phosphate transport system, permease component | -57.1 |
| *cg2925* | *pstS* | Enzyme II sucrose protein | -30.7 |
| *cg2713* | *dhaS* | NADP-dependent aldehyde dehydrogenase | -10.0 |
| *cg1791* | *gapA* | Glyceraldehyde-3-phosphate dehydrogenase | -8.3 |
| *cg2800* | *pgm* | Phosphoglucomutase | -8.0 |
| *cg3146* | *bglY* | Beta-glucosidase-fragment | -7.1 |
| *cg3219* | *ldh* | L-lactate dehydrogenase | -5.3 |
| *cg3218* | *-* | Pyruvate kinase-like protein | -5.1 |
| *cg1790* | *pgk* | Phosphoglycerate kinase | -4.8 |
| *cg0067* | *gabD3* | Succinate-semialdehyde dehydrogenase (NADP^+^) | -3.9 |
| *cg0973* | *pgi* | Glucose-6-phosphate isomerase | -3.7 |
| *cg3148* | *-* | ABC-type cobalamin transport system ATPase component | -3.5 |
| *cg2090* | *suhB* | MYO-inositol-1(or 4)-monophosphatase | -3.0 |
| *cg2953* | *xylC* | Benzaldehyde dehydrogenase | -2.8 |
| *cg2291* | *pyk* | Pyruvate kinase | -2.7 |
| *cg1789* | *tpiA* | Triosephosphate isomerase | -2.7 |
| *cg2987* | *dacB* | Penicillin-binding protein, D-Ala-D-Ala carboxypeptidase | -2.6 |
| *cg3068* | *fba* | Fructose-bisphosphate aldolase | -2.4 |
| *cg2558* | *-* | Related to aldose 1-epimerase | -2.2 |
| *cg0503* | *aroD* | Probable 3-dehydroquinate dehydratase (3-dehydroqu | -2.1 |
| **Pentose phosphate pathway** | | | |
| *cg2658* | *rpi* | Possible phosphopentose isomerase | 5.9 |
| *cg1546* | *rbsK1* | Putative ribokinase protein | 4.7 |
| *cg1157* | *glpX* | GLPX-like protein | 4.0 |
| *cg1776* | *tal* | Transaldolase | 3.0 |
| *cg1075* | *prsA* | Phosphoribosyl pyrophosphate synthase isozyme 2 pr | 2.6 |
| *cg0458* | *deoC* | Deoxyribose-phosphate aldolase | 2.2 |
| *cg2800* | *pgm* | Phosphoglucomutase | -8.0 |
| *cg0973* | *pgi* | Glucose-6-phosphate isomerase | -3.7 |
| *cg3068* | *fba* | Fructose-bisphosphate aldolase | -2.4 |
| *cg2609* | *valS* | Putative valine-trna ligase | -2.3 |
| **TCA cycle** | | | |
| *cg2836* | *sucD* | Succinyl-CoA synthetase alpha subunit | 53.2 |
| *cg2837* | *sucC* | Succinyl-CoA synthetase beta subunit | 8.3 |
| *cg1145* | *fumC* | Fumarate hydratase | 6.8 |
| *cg3359* | *trpE* | Anthranilate synthase component I | 5.9 |
| *cg3169* | *pck* | Probable phosphoenolpyruvate carboxykinase protein | 4.9 |
| *cg0949* | *gltA* | Citrate synthase | 4.4 |
| *cg0985* | *citE* | Citryl-CoA lyase beta subunit homolog | 4.4 |
| *cg0762* | *prpC2* | Methylcitrate synthase | 4.1 |
| *cg0653* | *rpsK* | Ribosomal protein S11 | 3.8 |
| *cg2421* | *aceF* | Dihydrolipoamide succinyltransferase | 3.5 |
| *cg0766* | *icd* | Isocitrate dehydrogenase | 2.5 |
| *cg2845* | *pstC* | Permease component of phosphate transport | -57.1 |
| *cg2846* | *pstS* | ABC-type phosphate transport system, secreted component | -30.7 |
| *cg1737* | *acn* | Aconitase | -3.9 |
| *cg2987* | *dacB* | Penicillin-binding protein, D-Ala-D-Ala carboxypeptidase | -2.6 |
| *cg2613* | *mdh* | Malate dehydrogenase oxidoreductase protein | -2.2 |
| *cg0503* | *aroD* | Probable 3-dehydroquinate dehydratase | -2.1 |
| *cg0798* | *prpC1* | (Methyl)citrate synthase | -2.1 |
| **Pyruvate metabolism** | | | |
| *cg2840* | *actA* | Butyryl-coa:acetate coenzyme a transferase | 33.3 |
| *cg3096* | *ald* | Aldehyde dehydrogenase | 24.0 |
| *cg3335* | *malE* | Malic enzyme | 8.3 |
| *cg2837* | *sucC* | Succinyl-coa synthetase beta subunit | 8.3 |
| *cg3169* | *pck* | Probable phosphoenolpyruvate carboxykinase protein | 4.9 |
| *cg2192* | *mqo* | Malate:quinone oxidoreductase oxidoreductase | 3.9 |
| *cg0653* | *rpsK* | Ribosomal protein S11 | 3.8 |
| *cg0303* | *leuA* | 2-Isopropylmalate synthase | 3.2 |
| *cg1656* | *ndh* | NADP dehydrogenase | 2.6 |
| *cg1253* | *dapC* | Succinyldiaminopimelate aminotransferase | 2.4 |
| *cg1583* | *argD* | Acetylornithine aminotransferase | 2.3 |
| *cg2845* | *pstC* | Permease component of phosphate transport | -57.1 |
| *cg2846* | *pstS* | ABC-type phosphate transport system, secreted component | -30.7 |
| *cg2713* | *dhaS* | NADP-dependent aldehyde dehydrogenase | -10.0 |
| *cg2891* | *poxB* | Pyruvate dehydrogenase | -8.9 |
| *cg3219* | *ldh* | L-lactate dehydrogenase | -5.3 |
| *cg3218* | *-* | Pyruvate kinase-like protein | -5.1 |
| *cg0498* | *hemC* | Porphobilinogen deaminase | -3.1 |
| *cg0071* | *-* | Metallo-beta-lactamase superfamily | -3.0 |
| *cg1856* | *-* | Zn-dependent hydrolase | -2.9 |
| *cg2953* | *xylC* | Benzaldehyde dehydrogenase | -2.8 |
| *cg2291* | *pyk* | Pyruvate kinase | -2.7 |
| *cg2987* | *dacB* | Penicillin-binding protein, D-Ala-D-Ala carboxypeptidase | -2.6 |
| *cg0141* | *-* | Glyoxalase/bleomycin resistance protein/dioxygenas | -2.6 |
| *cg0388* | *-* | Zn-dependent hydrolase | -2.3 |
| *cg2613* | *mdh* | Malate dehydrogenase oxidoreductase protein | -2.2 |
| *cg1482* | *-* | Zn-dependent hydrolases, including glyoxylases | -2.1 |
| *cg0503* | *aroD* | Probable 3-dehydroquinate dehydratase (3-dehydroqu) | -2.1 |
| **Branched-chain amino acid transportation** | | | |
| *cg2537* | *brnQ* | Branched-chain amino acid uptake carrier | -1.2 |
| *cg0315* | *brnE* | Branched chain amino acid exporter, small subunit | 4.0 |
| *cg0314* | *brnF* | Branched chain amino acid exporter, large subunit | 25.3 |
| *cg0313* | *lrp* | Leucine responsive regulation | -1.0 |
| **Elongation factors** | | | |
| *cg0583* | *fusA* | Elongation factor G | 2.8 |
| *cg0587* | *tuf* | Elongation factor Tu | 4.1 |
| *cg1123* | *greA* | Transcription elongation factor GreA | 5.1 |
| *cg1825* | *efp* | Translation elongation factor P | 1.7 |
| *cg2221* | *tsf* | Translation elongation factor Ts | 2.1 |
| **Ribosomal proteins** | | | |
| **Large subunit ribosomal protein** | | | |
| *cg0600* | *rplV* | Large subunit ribosomal protein L22 | 37.7 |
| *cg0610* | *rplE* | Large subunit ribosomal protein L5 | 30.5 |
| *cg0603* | *rpmC* | Large subunit ribosomal protein L29 | 23.2 |
| *cg0630* | *rplR* | Large subunit ribosomal protein L18 | 21.5 |
| *cg0609* | *rplX* | Large subunit ribosomal protein L24 | 21.4 |
| *cg0573* | *rplL* | Large subunit ribosomal protein L7/L12 | 20.9 |
| *cg0629* | *rplF* | Large subunit ribosomal protein L6 | 20.7 |
| *cg0602* | *rplP* | Large subunit ribosomal protein L16 | 19.1 |
| *cg0596* | *rplD* | Large subunit ribosomal protein L4 | 18.7 |
| *cg0632* | *rpmD* | Large subunit ribosomal protein L30 | 17.9 |
| *cg1565* | *rplT* | Large subunit ribosomal protein L20 | 16.4 |
| *cg0598* | *rplB* | Large subunit ribosomal protein L2 | 16.0 |
| *cg0634* | *rplO* | Large subunit ribosomal protein L15 | 14.1 |
| *cg1564* | *rpmI* | Large subunit ribosomal protein L35 | 12.6 |
| *cg0608* | *rplN* | Large subunit ribosomal protein L14 | 11.3 |
| *cg0656* | *rplQ* | Large subunit ribosomal protein L17 | 11.0 |
| *cg0572* | *rplJ* | Large subunit ribosomal protein L10 | 10.2 |
| *cg0564* | *rplA* | Large subunit ribosomal protein L1 | 7.4 |
| *cg3306* | *rplI* | Large subunit ribosomal protein L9 | 7.2 |
| *cg0594* | *rplC* | Large subunit ribosomal protein L3 | 6.2 |
| *cg0990* | *rpmG* | Large subunit ribosomal protein L33 | 5.2 |
| *cg1072* | *rplY* | Large subunit ribosomal protein L25 | 4.7 |
| *cg2235* | *rplS* | Large subunit ribosomal protein L19 | 4.0 |
| *cg0597* | *rplW* | Large subunit ribosomal protein L23 | 3.6 |
| *cg0991* | *rpmB* | Large subunit ribosomal protein L28 | 3.3 |
| *cg0563* | *rplK* | Large subunit ribosomal protein L11 | 3.2 |
| *cg0673* | *rplM* | Large subunit ribosomal protein L13 | 2.4 |
| *cg0994* | *rpmE* | Large subunit ribosomal protein L31 | 2.1 |
| *cg0995* | *rpmF* | Large subunit ribosomal protein L32 | 2.0 |
| *cg2594* | *rpmA* | Large subunit ribosomal protein L27 | 1.1 |
| *cg2595* | *rplU* | Large subunit ribosomal protein L21 | 1.0 |
| *cg2791* | *rpmJ* | Large subunit ribosomal protein L36 | -2.9 |
| **Small subunit ribosomal protein** | | | |
| *cg0599* | *rpsS* | Small subunit ribosomal protein S19 | 21.2 |
| *cg0582* | *rpsG* | Small subunit ribosomal protein S7 | 20.0 |
| *cg0631* | *rpsE* | Small subunit ribosomal protein S5 | 17.8 |
| *cg0604* | *rpsQ* | Small subunit ribosomal protein S17 | 16.9 |
| *cg0581* | *rpsL* | Small subunit ribosomal protein S12 | 8.2 |
| *cg2253* | *rpsP* | Small subunit ribosomal protein S16 | 7.6 |
| *cg2167* | *rpsO* | Small subunit ribosomal protein S15 | 5.9 |
| *cg2573* | *rpsT* | Small subunit ribosomal protein S20 | 4.9 |
| *cg0654* | *rpsD* | Small subunit ribosomal protein S4 | 4.3 |
| *cg0628* | *rpsH* | Small subunit ribosomal protein S8 | 4.0 |
| *cg0988* | *rpsR* | Small subunit ribosomal protein S18 | 3.9 |
| *cg0653* | *rpsK* | Small subunit ribosomal protein S11 | 3.8 |
| *cg0989* | *rpsN* | Small subunit ribosomal protein S14 | 3.3 |
| *cg0593* | *rpsJ* | Small subunit ribosomal protein S10 | 3.1 |
| *cg0674* | *rpsI* | Small subunit ribosomal protein S9 | 2.5 |
| *cg1531* | *rpsA* | Small subunit ribosomal protein S1 | 2.5 |
| *cg0601* | *rpsC* | Small subunit ribosomal protein S3 | 2.4 |
| *cg0652* | *rpsM* | Small subunit ribosomal protein S13 | 1.8 |
| *cg2222* | *rpsB* | Small subunit ribosomal protein S2 | 1.7 |
| *cg3308* | *rpsF* | Small subunit ribosomal protein S6 | 1.1 |
| **Cell division and cell wall synthesis** | | | |
| *cg0915* | *ftsX* | Cell division protein | 15.8 |
| *cg0914* | *ftsE* | Cell division ATP-binding protein | 5.7 |
| *cg0001* | *dnaA* | Chromosomal replication initiator protein | 3.6 |
| *cg2366* | *ftsZ* | Cell division GTPase | 3.5 |
| *cg2367* | *ftsQ* | Cell division septal protein | 3.4 |
| *cg2375* | *ftsI* | Cell division protein FtsI | 2.9 |
| *cg1610* | *parA2* | Putative ATPase involved in chrosome partitioning | 2.9 |
| *cg2378* | *mraZ* | Cell division protein MraZ | 2.6 |
| *cg2377* | *mraW* | S-adenosylmethionine-dependent methyltransferase | 2.3 |
| *cg2620* | *clpX* | Putative ATP-dependent protease | 2.2 |
| *cg2370* | *ftsW* | Bacterial cell division membrane protein | -4.6 |
| *cg3304* | *dnaB* | Putative replicative DNA helicase | -3.6 |
| **Sigma factors** | | | |
| *cg2092* | *sigA* | RNA polymerase sigma 70 factor | 3.5 |
| *cg1271* | *sigE* | Putative RNA polymerase sigma factor | 2.7 |
| *cg0309* | *sigC* | Sigma-70 factor (ECF subfamily) | 1.1 |
| *cg3420* | *sigM* | RNA polymerase sigma-70 factor, ECF subfamily | -2.6 |
| *cg0696* | *sigD* | Putative RNA polymerase sigma factor | -2.2 |
| *cg0876* | *sigH* | Putative RNA polymerase sigma factor | -1.2 |
| *cg2102* | *sigB* | RNA polymerase sigma factor | -1.2 |
| **ABC transporters** | | | |
| *cg0926* | *-* | ABC-type cobalamin transport permease component | 10.6 |
| *cg2136* | *gluA* | Glutamate uptake system ATP-binding protein | 8.6 |
| *cg2138* | *gluC* | Glutamate permease | 5.2 |
| *cg1762* | *sufC* | Iron-regulated ABC transporter ATPase subunit | 4.4 |
| *cg0927* | *-* | ABC-type cobalamin permease component | 4.2 |
| *cg0767* | *-* | Siderophore-interacting protein | 3.0 |
| *cg3129* | *-* | ABC-type transport system, ATPase component | 2.6 |
| *cg0748* | *-* | cobalamin transport systems, secreted component | 2.6 |
| *cg1405* | *-* | Siderophore-interacting protein | 2.5 |
| *cg0771* | *-* | DTXR/iron regulated lipoprotein precursor, secrete | 2.5 |
| *cg0928* | *-* | ABC-type cobalamin transport system, ATPase component | 2.5 |
| *cg0924* | *-* | ABC-type cobalamin/Fe3+-siderophores transport sys | 2.3 |
| *cg2139* | *gluD* | Glutamate permease | 2.1 |
| *cg2137* | *gluB* | Glutamate secreted binding protein | 1.3 |
| *cg2843* | *pstB* | ABC-type phosphate transport system, ATPase component | 114.8 |
| *cg1569* | *ugpE* | sn-glycerol-3-phosphate transport system permease protein | 114.4 |
| *cg2844* | *pstA* | ABC-type phosphate transport permease component | -71.3 |
| *cg1568* | *ugpA* | sn-Glycerol-3-phosphate transport system permease protein | -60.0 |
| *cg2845* | *pstC* | ABC-type phosphate transport permease component | -57.1 |
| *cg2846* | *pstS* | ABC-type phosphate transport system, secreted component | -30.7 |
| *cg1571* | *ugpC* | ABC-type sugar transport systems, ATPase component | -14.3 |
| *cg3367* | *-* | ABC-type multidrug transport system, ATPase component | -13.9 |
| *cg1570* | *ugpB* | secreted sn-glycerol-3-phosphate-binding protein | -11.3 |
| *cg3368* | *-* | ABC-transporter permease protein | -5.8 |
| *cg1082* | *-* | putative membrane protein | -5.2 |
| *cg1647* | *-* | ABC-type multidrug transport permease component | -2.1 |

^a^Factors of Regulation are shown as the ratio of gene RPKM of VWB-1 versus the gene RPKM of ATCC 13869, “Regulation>0” represents gene up-regulation and “Regulation<0” represents gene down-regulation. DEGs with FDR ≤ 0.001 are regarded as credible.
